# Static magnetic field-induced IL-6 secretion in periodontal ligament stem cells accelerates orthodontic tooth movement

**DOI:** 10.1038/s41598-024-60621-6

**Published:** 2024-04-29

**Authors:** Shitong Luo, Zhilian Li, Lizhiyi Liu, Juan Zhao, Wenbin Ge, Kun Zhang, Zhi Zhou, Yali Liu

**Affiliations:** 1 Department of Orthodontics, School and Hospital of Stomatology, Kunming Medical University, 1088 Middle Haiyuan Road, High-Tech Zone, Kunming, 650106 Yunnan China; 2 Yunnan Key Laboratory of Stomatology, Kunming, 650106 China; 3 Department of Orthodontics, Suining Central Hospital, Suining, 629000 China; 4 Department of Pathology, Suining Central Hospital, Suining, 629000 China; 5 Department of Orthodontics, Affiliated Hospital of Yunnan University, Yunnan University, 176 Qingnian Road, Wuhua District, Kunming, 650021 Yunnan China

**Keywords:** Tooth movement, Static magnetic field, IL-6, Osteoclast, Acceleration, Oral diseases, Mesenchymal stem cells, Materials science

## Abstract

Static magnetic field (SMF) promoting bone tissue remodeling is a potential non-invasive therapy technique to accelerate orthodontic tooth movement (OTM). The periodontal ligament stem cells (PDLSCs), which are mechanosensitive cells, are essential for force-induced bone remodeling and OTM. However, whether and how the PDLSCs influence the process of inflammatory bone remodeling under mechanical force stimuli in the presence of SMFs remains unclear. In this study, we found that local SMF stimulation significantly enhanced the OTM distance and induced osteoclastogenesis on the compression side of a rat model of OTM. Further experiments with macrophages cultured with supernatants from force-loaded PDLSCs exposed to an SMF showed enhanced osteoclast formation. RNA-seq analysis showed that interleukin-6 (IL-6) was elevated in force-loaded PDLSCs exposed to SMFs. IL-6 expression was also elevated on the pressure side of a rat OTM model with an SMF. The OTM distance induced by an SMF was significantly decreased after injection of the IL-6 inhibitor tocilizumab. These results imply that SMF promotes osteoclastogenesis by inducing force-loaded PDLSCs to secrete the inflammatory cytokine IL-6, which accelerates OTM. This will help to reveal the mechanism of SMF accelerates tooth movement and should be evaluated for application in periodontitis patients.

## Introduction

As one of the three oral diseases with the highest incidence, malocclusion seriously endangers oral function and patients' physical and mental health^1^. Orthodontic treatment, which changes the position of teeth through tooth movement, is effective for malocclusion. Orthodontic tooth movement (OTM) is a “sterile” inflammatory process that involves balanced tension-side bone growth and pressure-side bone resorption. Moreover, osteoclasts are in charge of the bone resorption necessary to create space for OTM^2^. Currently, comprehensive orthodontic treatment takes an average duration of 18–36 months. The long treatment period is not only time-consuming and costly for patients, but also greatly increases the risk of complications such as white spots on the tooth surface, dental caries, and poor periodontal health^3^. Therefore, accelerating tooth movement and reducing treatment time is an urgent unmet need in modern orthodontic treatment.

The static magnetic field (SMF) is a physical therapy widely used to treat and prevent many different disorders. In the patient with arthritis, SMF helps relieve pain and increases bone density in the joints^4,5^. Magnets have been used in oral clinical practice for many years. Magnetic orthodontic appliances can promote bone remodeling, adjust tooth and jaw position, and accelerate OTM^6–8^. SMF is regarded to be competent in regulating the inflammatory tissue remodeling process. SMF inhibits inflammation and can promote the subsidence of inflammation to accelerate wound healing^9^. SMF also attenuates levels of lipopolysaccharide (LPS)-induced inflammation in dental pulp stem cells^10^. Moreover, researchers found that SMF can promote the secretion of inflammatory factor IL-6 from microglia^11^. The OTM process takes place in an aseptic inflammatory microenvironment, characterized by increased inflammatory cytokine and chemokine production and enhanced inflammatory immune cell activation. SMF generated by a magnet has been gradually acknowledged as an effective factor in accelerating OTM. Thus, whether SMF mediates accelerated OTM by affecting inflammation levels is worthy of further investigation.

Periodontal tissue is the primary tissue stimulated by mechanical force during tooth movement. Periodontal ligament stem cells (PDLSCs), the primary mesenchymal stem cells of the periodontal tissues, are mechanosensitive cells that support the inflammation and bone reconstructing process during OTM^12,13^. Recent reports demonstrate that PDLSCs express gas molecules, chemokines, and inflammatory cytokines to regulate OTM’s inflammatory bone reconstructing process^14,15^. Osteoblasts, osteoclasts, and osteocytes, driven by inflammation and hormones, sustain bone remodeling^16^. Inflammatory cytokines, such as tumor necrosis factor-alpha (TNF-α) and interleukin-6 (IL-6), enhance macrophage activation and antigen presentation, inducing osteoclast formation and regulating inflammation through different mechanisms^17,18^. IL-6 is assumed to be the “classic” pro-inflammatory bone-resorption cytokine. Moreover, researchers have discovered that IL-6 can increase osteoclastogenesis by signaling to osteoclast precursors and encouraging the osteoblast lineage to express RANKL^19,20^. Our previous research has revealed a significant regulatory effect of SMF on PDLSC function^21^. However, whether the IL-6 secreted by the PDLSCs under stimulation by SMF accelerates OTM requires further exploration.

Given the importance of IL-6 in SMF-induced acceleration of tooth movement, we hypothesized that the IL-6 secreted by PDLSCs under SMF stimulation influences osteoclast formation and alveolar bone remodeling, thereby accelerating OTM. This study aimed to investigate whether and how SMF modulates osteoclast formation, which promotes periodontal inflammatory bone remodeling. The findings of this experiment can advance the understanding of the molecular mechanism of SMF-induced acceleration of OTM and guide the orthodontic clinical application of magnetic materials.

## Results

### Establishment of the local magnetic field-exposed rat model of OTM

**Figure 1:**
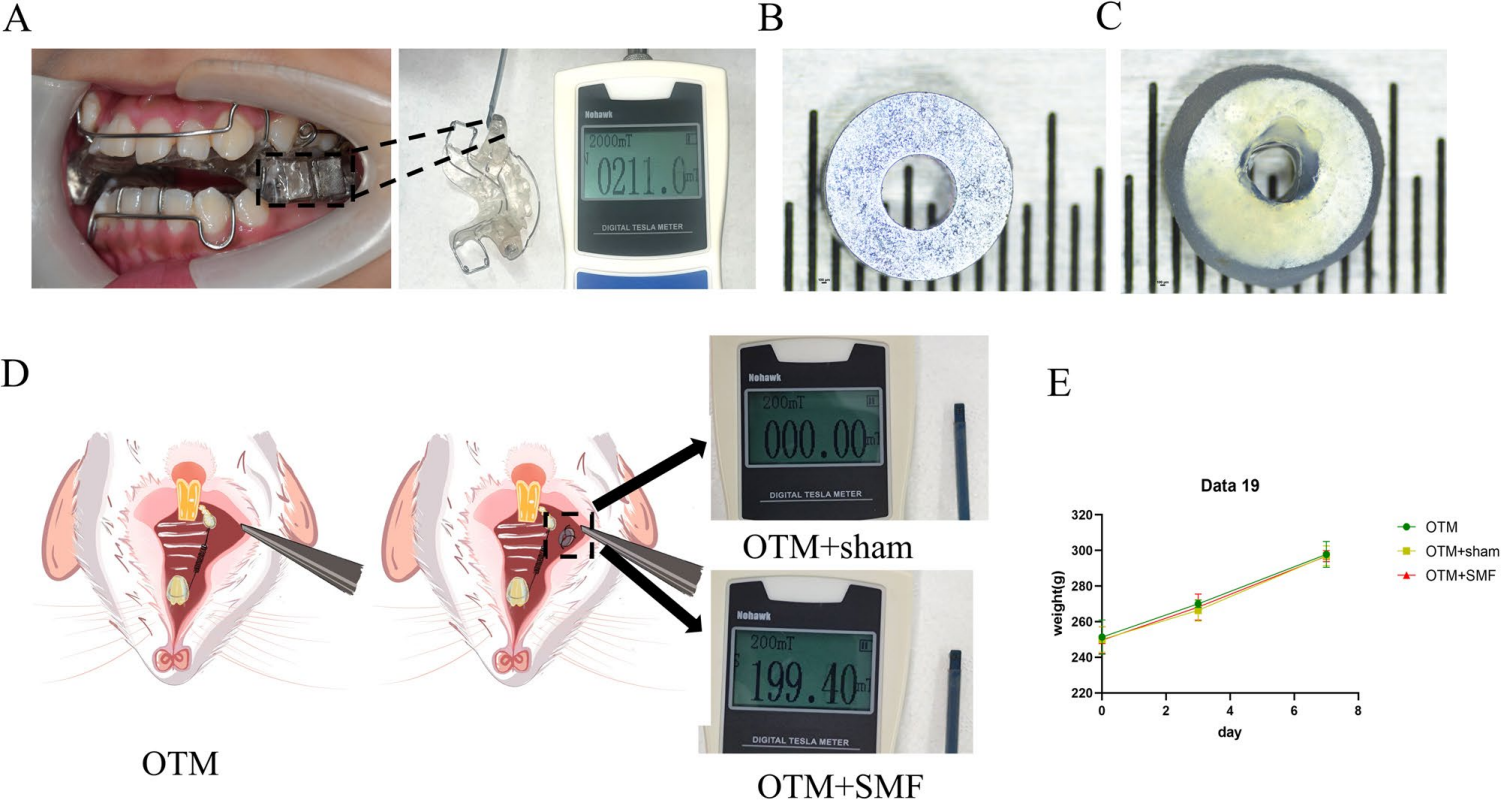
Established local magnetic field exposure of tooth movement in the rat model. (**A**) The clinical magnetic orthodontic appliance and its magnetic field strength. (**B**) Size of the customized magnet. (**C**) Size of the biosafety resin-coated magnet. (**D**) Modeling approaches in the OTM group, OTM + sham group, and OTM + SMF group. (**E**) Body-weight curves of rats in each group over 7 d.

To simulate clinical magnetic orthodontics appliances (Fig. S1), we measured the surface magnetic field strength of magnetic appliances used clinically (200 ± 50 mT; Fig. 1A). The customized, small magnetic-field exposure magnet we used had a diameter of 4 mm and a hole of diameter 1.5 mm in the center (Fig. 1B). The magnet was manually covered with a resinous material having good biological safety to reduce biological toxicity (Fig. 1C). The surface strength of the custom-made magnets was 200 ± 20 mT (Fig. 1D), and the magnetic field surface strength of the dummy magnets used in the sham surgical treatment was 0 ± 0.02 mT (Fig. 1D). The sutures were adequate to establish a stable local magnetic field-exposure model (Fig. 1D). We observed no significant effect of surgery on mastication or the body weight of the experimental animals (Fig. 1E).

### SMF accelerated OTM in rats

**Figure 2:**
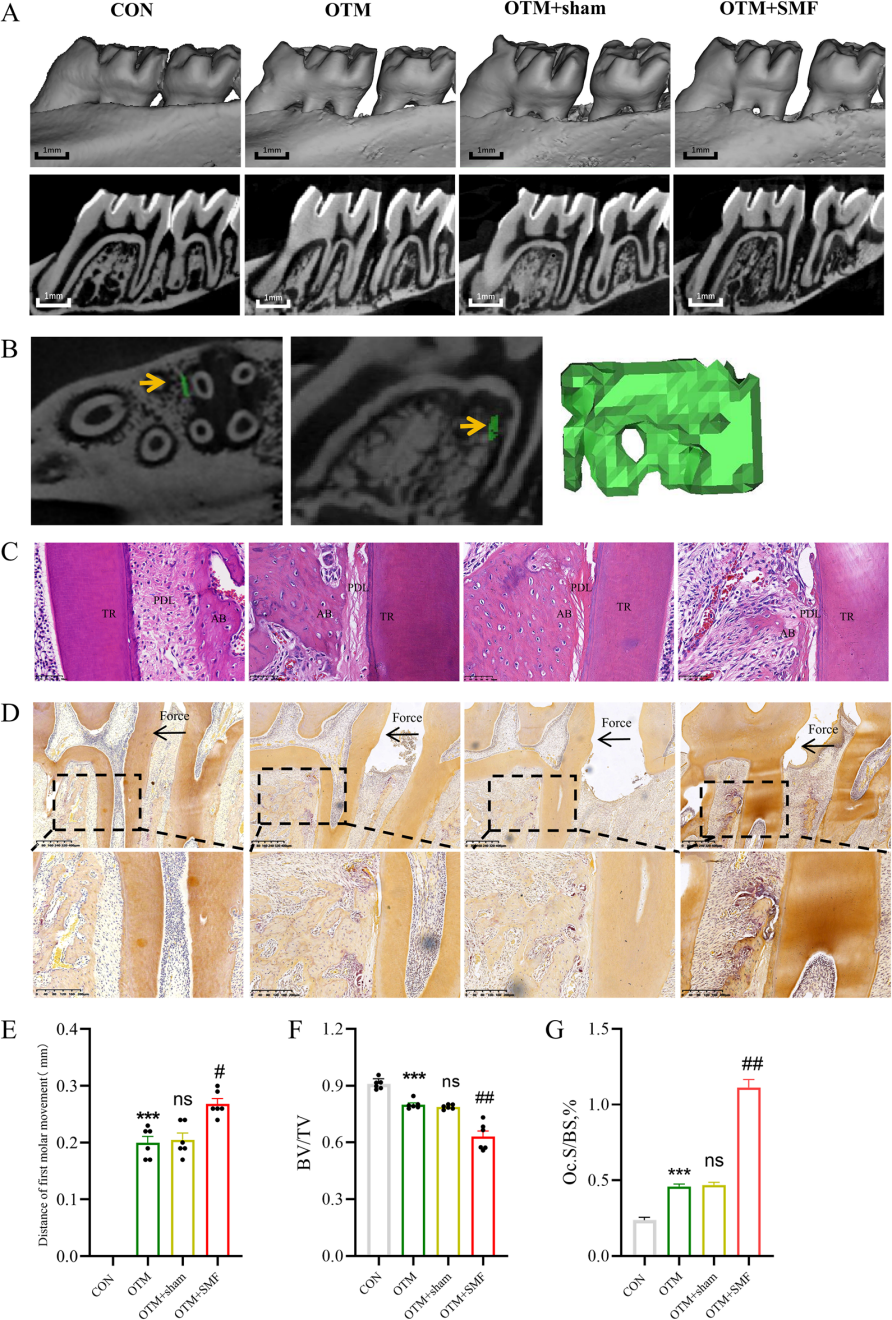
SMF promoted tooth movement in rats. (**A**,**E**) Micro-CT images for measurement and analysis of first molar movement distance after 7 days; scale bar: 1 mm, n = 6. (**B**,**F**) Bone volume fraction (BV/TV) of the pressure side; the measurement areas are shown in green area of (**B**). (**C**) HE stain of four groups; *TR* tooth root, *PDL* periodontal ligament, *AB* alveolar bone. (**D**,**G**) Representative TRAP images of the pressure side, n = 3; osteoclast surface per bone surface (Oc.S/B.S%). All data are mean ± SD, ns, not significant vs OTM; ***P < 0.001 vs Con group; ^#^P < 0.05 vs OTM + sham; ^##^P < 0.01 vs OTM + sham.

To detect changes in tooth-movement distances in the rats 7 days after surgery, we scanned the alveolar bone of the euthanized rats by Micro-CT and measured the tooth-movement distances of each group (Fig. 2A). We found no significant difference between the tooth movement distance of 0.21 ± 0.03 mm observed in the OTM + sham group and the 0.20 ± 0.024 mm observed in the OTM group, suggesting that the surgical operation of suturing the local magnet had no significant effect on tooth movement. The OTM + SMF group presented much more movement than did the OTM + sham group (0.27 ± 0.023 vs 0.21 ± 0.03 mm, P < 0.05; Fig. 2E), with an approximately 29% increase. We measured the bone volume fraction (bone volume/total volume, BV/TV; Fig. 2B) in the alveolar bone on the pressure side of OTM and found a considerable decrease (P < 0.05) in the OTM + SMF group (Fig. 2F). It was suggested that the promotion of tooth movement by SMF may be associated with bone resorption. In addition, the HE-stained sections showed the tissue space of periodontal membrane was uniform, the fibers arranged neatly and the fibroblast distributed evenly in main fiber in control group, while the periodontal membrane width on the pressure side were shortened and the fibers were compressed in other three groups (Fig. 2C). The results revealed that SMF could accelerate tooth movement in rats.

### SMF promoted the formation of pressure-side osteoclasts

Because resorption of the alveolar bone of pressure-side is the rate-limiting step of OTM, we studied the distribution and area of the osteoclasts on TRAP-stained tissue sections. TRAP-positive cells were significantly more densely distributed near the periodontal ligament on the pressure side than in other areas (Fig. 2D). We found no significant difference in percent osteoclast surface per unit of bone surface (Oc.S/B.S,%) between the OTM group and the OTM + sham group, but the Oc.S/B.S,% in the OTM + SMF group was substantially higher than that in the OTM + sham group (P < 0.01) (Fig. 2G), suggesting that SMF promoted the formation of osteoclasts on the pressure side, which may be the reason for accelerated tooth movement.

### SMF promoted the ability of PDLSCs under mechanical pressure to induce the differentiation of BMMs into osteoclasts

**Figure 3:**
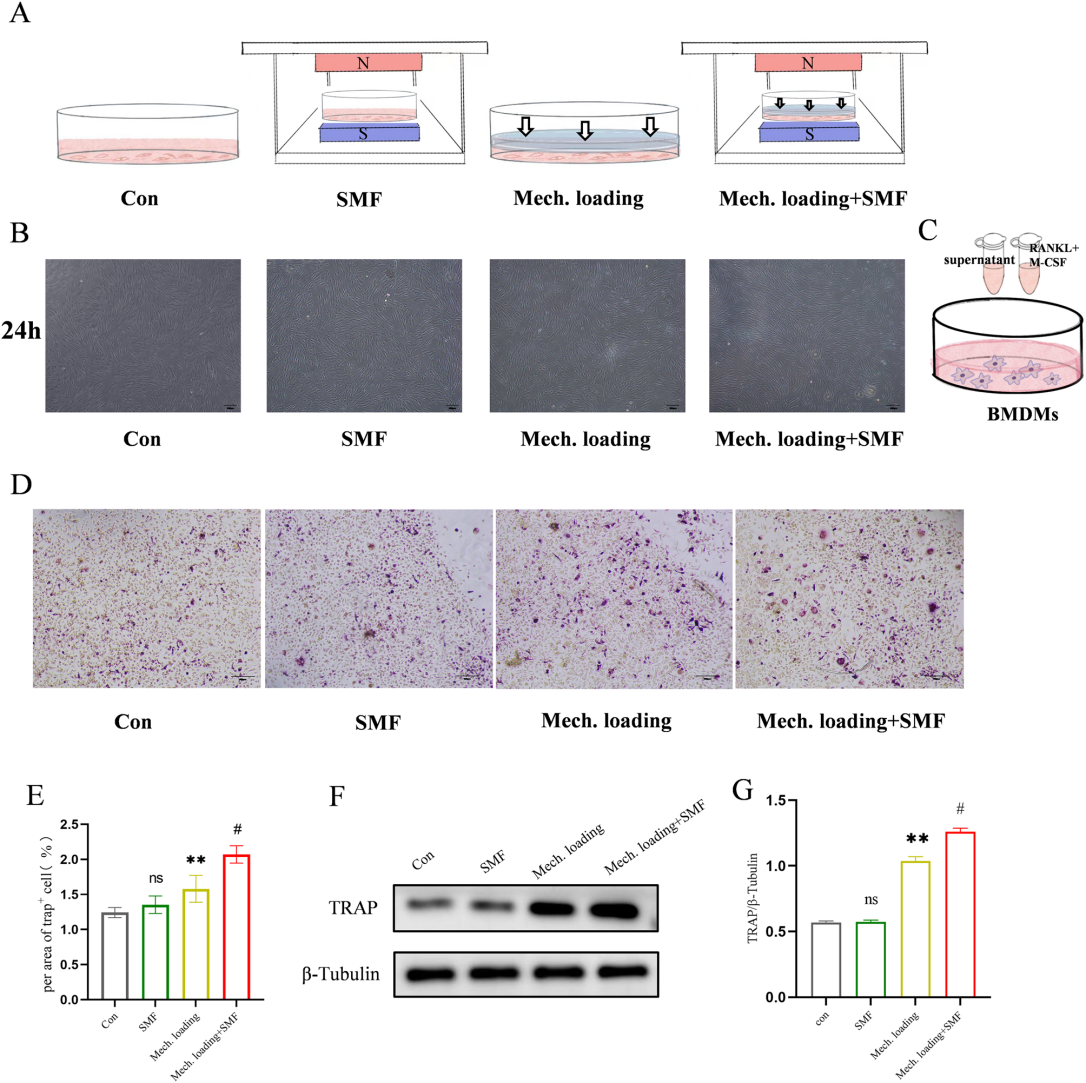
SMF promoted osteoclast formation by PDLSCs under mechanical stress. (**A**) Schematic diagram of the in-vitro models. (**B**) Morphology of PDLSCs stimulated by mechanical loading and SMF after 24 h. (**C**) Schematic diagram of osteoclast formation induced by the PDLSCs supernatant. (**D**,**E**) Representative TRAP images showing osteoclast formation after 7 days of induction. (**F**,**G**) Western blot image and semi-quantitative measurement of TRAP expression in osteoclasts after 7 days of induction. n = 3; ns, not significant; **P < 0.01 vs Con group; ^#^P < 0.05 vs Mech. loading group.

PDLSCs are important effector cells for tooth movement. To further verify that SMF promotes the induction of osteoclast formation by pressure-loaded PDLSCs, we cultured four groups of PDLSCs: Control group in normal conditions; SMF group exposed to SMF of strength approximately 200 ± 20 mT; Mech. loading group with in vitro mechanical loading stimulation of 2 g/cm^2^; and Mech. loading + SMF group combined with Mech. Loading and SMF (Fig. 3A). The PDLSCs in this study have been identified (Fig. S2). After 24 h, there was no significant difference in the morphology of PDLSCs stimulated by mechanical loading and SMF (Fig. 3B). PDLSCs’ supernatant combined with the osteoclast-inducible medium induced osteoclast-differentiation in bone-marrow macrophages (Fig. 3C). After 7 days of osteoclast differentiation experiments using PDLSC supernatant, the TRAP staining showed the appearance of osteoclasts in each group (Fig. 3D,E). The negative control had almost no TRAP-positive osteoclasts (Fig. S3). The osteoclasts in the Mech. loading + SMF group were more than that in the Mech. loading group (P < 0.01) (Fig. 3D). TRAP protein levels in the osteoclasts of each group were determined by WB, and the outcomes revealed that the TRAP protein level in the Mech. loading + SMF group was considerably higher than that in the Mech. loading group (P < 0.01) (Fig. 3F,G), which was coherent with the outcomes of TRAP staining. These findings indicate that SMF stimulation enhanced the ability of PDLSCs to induce osteoclast formation under pressure stimulation.

### Transcriptomic analysis

**Figure 4:**
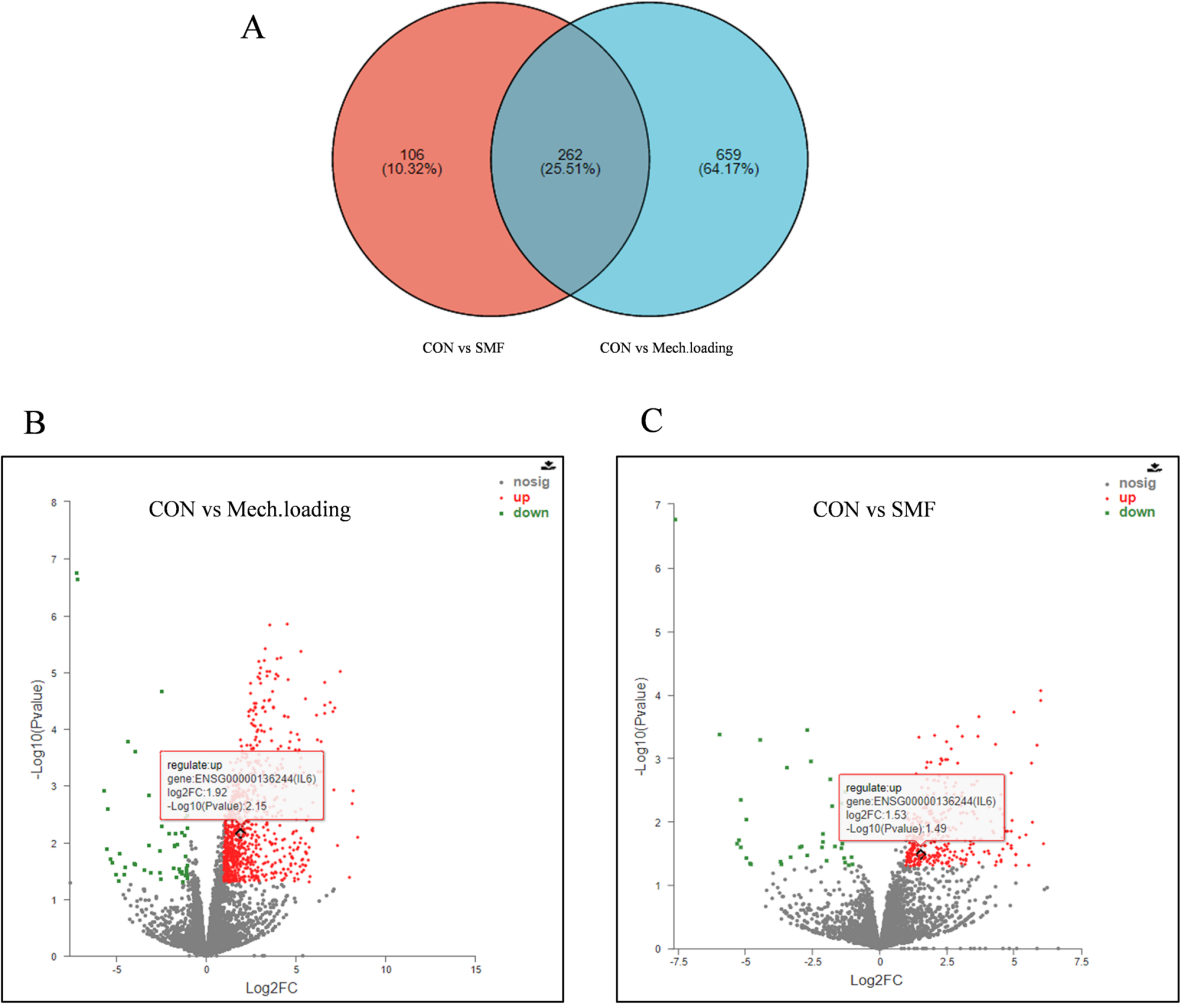
Transcriptomic analysis. (**A**) A Venn diagram shows the intersection of DEGs between the Mech. Loading group vs the Con group and the SMF group vs the Con group. (**B**) Volcano map of DEG in CON group vs Mech. loading group. (**C**) Volcano map of DEG in CON group vs SMF group. The red box shows the data information of the differentially expressed gene IL-6.

To further investigate the mechanism by which SMF promotes the induction of osteoclast formation by mechanically pressurized PDLSCs, we performed RNA-seq on each PDLSC group. The SMF group had 171 up-regulated genes and 39 down-regulated genes compared to the Con group. The Mech. loading group had 1184 up-regulated genes and 171 down-regulated genes compared to the Con group. The Mech. loading + SMF group showed 171 up-regulated genes and 39 down-regulated genes compared to the Mech. loading group (Fig. S5). Compared with the Con group, the intersection of DEGs in the SMF and Mech. loading groups was obtained using Venn, and 262 genes were found in Venn (Fig. 4A). We found that SMF upregulated the expression level of IL-6 in PDLSC, and mechanical stress also increased the expression level of IL-6 (Fig. 4B,C). Clinical and basic studies have shown that IL-6 is important in tooth movement and is closely related to bone reconstruction^22,23^. To further explore the change of IL-6 gene expression level, by analyzing the original sequencing data, we discovered that the Mech. loading + SMF group had higher levels of IL-6 expression than Mech. loading + sham group (Fig. S6). These findings implied that IL-6 might play a significant role in how SMF promoted OTM.

### SMF promoted IL-6 secretion by mechanically loaded PDLSCs

**Figure 5:**
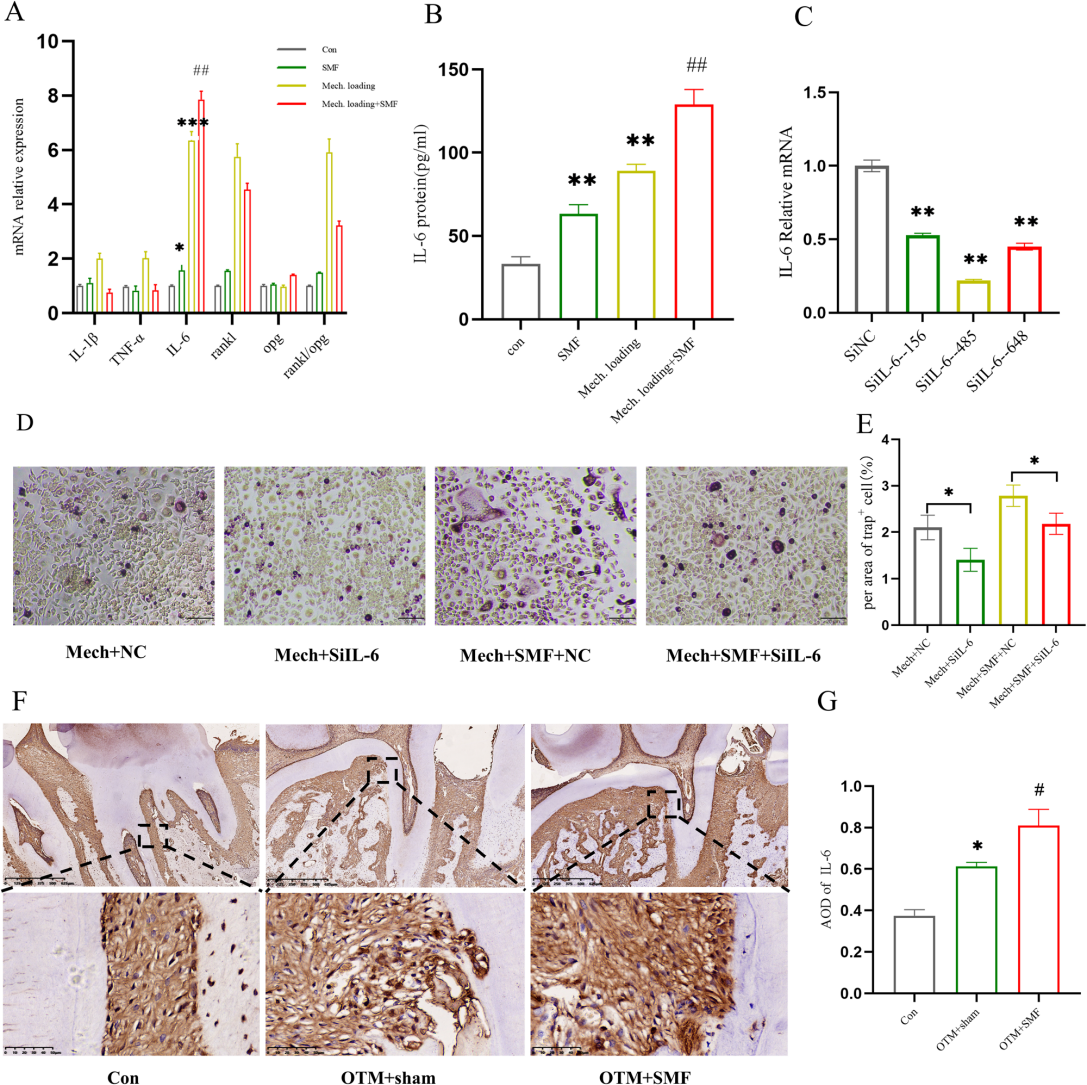
SMF promoted IL-6 secretion from PDLSCs under mechanical stress. (**A**) RT-PCR assay of expression levels of IL-1β, TNF-α, IL-6, RANKL, OPG, and RANKL/OPG relative to GAPDH in PDLSCs after 24 h. Data for each gene was processed using the 2ΔΔCt method. (**B**) ELISA of IL-6 protein in PDLSCs supernatant after 24 h. (**C**) RT-PCR was performed to evaluate the efficiency of IL-6 inhibition (*P < 0.05). (**D**,**E**) Representative TRAP images showing osteoclast formation after seven days of induction. (**F**,**G**) Immunohistochemistry of IL-6 expression levels on the pressure side of tooth movement. *AOD* integrated optical density/area; n = 3; data are mean ± SD; *P < 0.05 vs Con; ^#^P < 0.05 vs OTM + sham.

To verify the sequencing results and assess whether IL-6 is important for the induction of osteoclast development by PDLSCs in the presence of stress and SMF, RT-qPCR was used to assess the mRNA expression levels of IL-1β, TNF-α, IL-6, RANKL, OPG, and RANKL/OPG in the above four groups of PDLSCs after 24 h. The results showed that the SMF suppressed the mRNA expressions of the inflammatory factors TNF-α, IL-1β, and RANKL/OPG. However, IL-6 mRNA expression was considerably higher in the SMF and Mech. Loading groups than in the control group. The mRNA expression of IL-6 in the Mech. loading + SMF group was significantly higher than that in Mech. Loading group (P < 0.01) (Fig. 5A). This indicates that IL-6 may be a key factor involved in the acceleration of OTM by SMF. The quantity of IL-6 protein in the supernatants of PDLSCs cultured under various conditions were detected using ELISA, and the results showed the Mech. loading + SMF group had a significantly higher IL-6 levels than the Mech. loading group (P < 0.05; Fig. 5B). Inhibition of the expression level of IL-6 mRNA after transfection knockdown of IL-6 and stressing for 24 h using PCR showed that siIL6-485 was the most effective (P < 0.05; Fig. 5C). And the siIL-6 485 group had the lowest ability to induce osteoclasts (Fig. S4). Therefore, siIL6-485 was selected for subsequent experiments. Induction of osteoclast formation by PDLSC-conditioned medium with IL-6 inhibition under magnetic field and pressure treatment showed lower osteoclasts in the Mech. loading + SMF + siIL6 group than in the Mech. loading + SMF + siNC group (P < 0.05; Fig. 5D,E). Immunohistochemistry for IL-6 showed that positive cells were clustered near the periodontal membrane on the pressure side (Fig. 5F), and the IL-6 expression level was higher in the OTM + SMF group than in the OTM + sham group (p < 0.05) (Fig. 5G). These results suggest that SMFs promote osteoclast formation by promoting the secretion of IL-6 by PDLSCs under pressure, thus accelerating tooth movement.

### Tocilizumab attenuated the effect of SMF in promoting OTM

**Figure 6:**
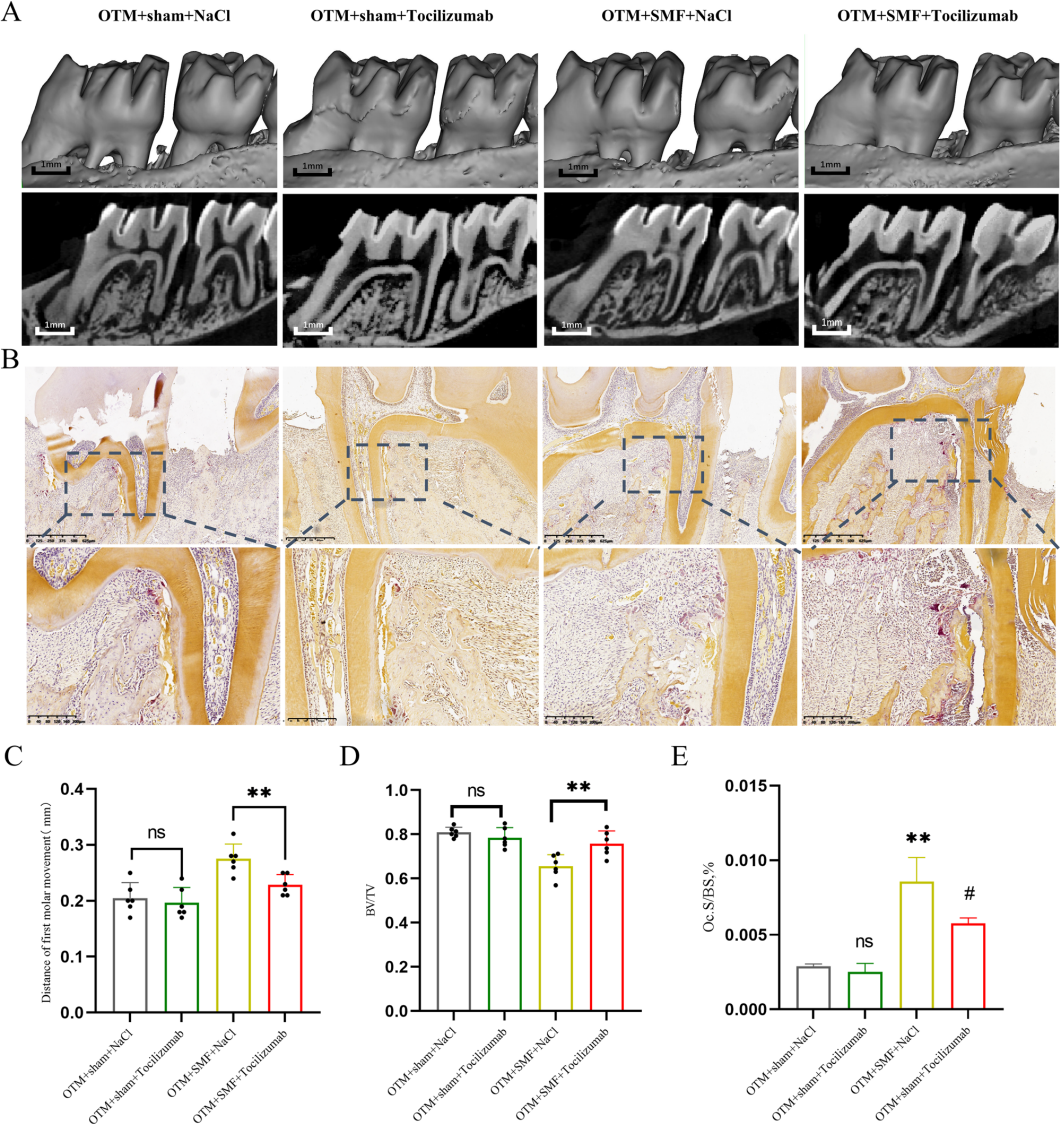
Tocilizumab reduced the effect of SMF in promoting OTM. (**A**,**C**) Micro-CT images were used to measure and analyze first-molar movement distance with inhibitor intervention after 7 days. Scale bar = 1 mm; n = 6. (**B**,**E**) Representative TRAP images on the pressure side. n = 3; Oc.S/B.S, % percent osteoclast surface per unit of the bone surface. (**D**) The bone volume fraction (BV/TV) is shown on the pressure side, with the measurement areas shown in the inset in (**A**). All data are mean ± SD; ns, not significant vs OTM + sham + NaCl; ^#^P < 0.05 vs OTM + SMF + NaCl.

**Figure 7:**
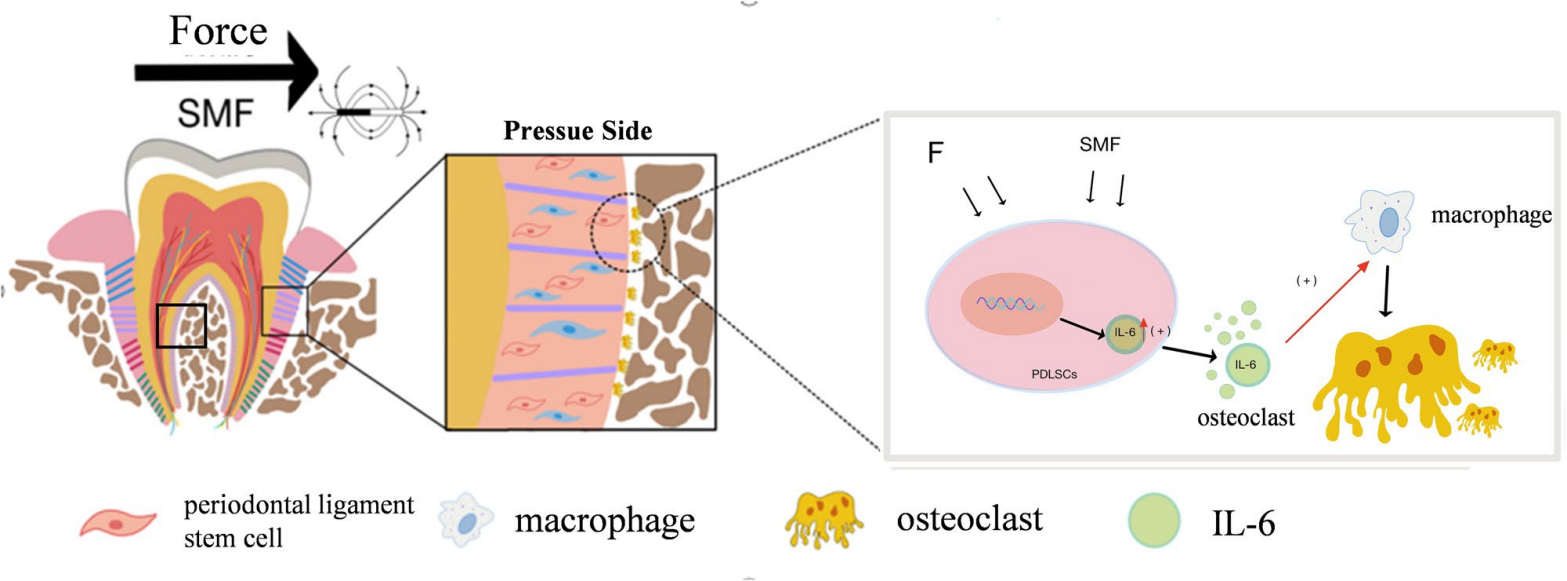
Schematic diagram of the mechanism by which SMF enhances tooth movement.

To further verify IL-6 as a key factor in promoting the effect of SMF in accelerating OTM, the IL-6 inhibitor tocilizumab was injected locally into rat periodontal tissues. After 7 days of tocilizumab treatment, the distance of tooth movement was analyzed using Mirco-CT. Tocilizumab has no significant effect on the tooth movement distance of sham-operated rats but has a significant effect on the tooth movement distance of the rats treated with a SMF. Compared with 0.28 ± 0.023 mm in the OTM + SMF + NaCl group, the tooth movement distance in the OTM + SMF + tocilizumab group was significantly reduced by 0.23 ± 0.015 mm (P < 0.05), indicating that the efficiency of SMF in promoting tooth movement was significantly inhibited; however, it was still slightly higher than that of the OTM + NaCl group by 0.21 ± 0.03 mm (Fig. 6A,C). The outcomes of the unit bone volume fraction (BV/TV) on the pressure side of the tooth revealed that tocilizumab decreased bone resorption of the pressure side, particularly in the SMF-stimulated rats (P < 0.05; Fig. 6D). TRAP staining and semi-quantitative analysis of the OC.S/B.S% of the osteoclasts showed that osteoclast formation was lower in the OTM + SMF + tocilizumab group than in the OTM + SMF + NaCl group (P < 0.05). There was no significant difference in osteoclasts between the OTM + sham + tocilizumab group and the OTM + sham + NaCL group (Fig. 6B,E). These results demonstrated that the IL-6 inhibitor tocilizumab attenuated the effect of SMF in promoting tooth movement by reducing osteoclast formation and action. The mechanism of SMF facilitation of tooth movement revealed by these results is shown schematically in Fig. 7.

## Discussion

Many auxiliary methods of accelerating tooth movement have been reported, which can be divided into surgical stimulation and physical stimulation according to the different ways of application. The use of invasive procedures to accelerate OTM has shown clear clinical efficacy, but the pain and infection associated with the procedure is an important limitation of its clinical application^24^. Physical stimulation is more acceptable to patients for its non-invasive and high safety characteristics. The main methods include magnetic field, laser, vibration, ultrasound. It is believed that the main reason to limit the speed of tooth movement is the osteoclastic activity on the pressure side^2^, so these therapies accelerate the reconstruction of periodontal bone tissue by promoting osteoclast formation. Some studies have shown that vibration can cooperate with pressure to stimulate PDL cells to secrete RANKL, PGE2, IL-6, IL-8, IL-1β, TNF-α and other factors to promote osteoclast formation^25–27^. Because the laser is blocked by the tissue and cannot penetrate the periodontal tissue directly to the periodontal ligament and bone tissue, the current research is limited to the gingival tissue, and it is found that the laser promotes tooth movement mainly by promoting RNAKL, IL-6, IL-8, COX-2, TNF-α and IL-1β in the gingival tissue^28–32^. The molecular mechanism of the ultrasonic and SMF is rarely reported, which only found that the ultrasonic and SMF continuously promote osteoclast formation and accelerate the rate of tooth movement^33,34^. The SMF has more advantages than other physical acceleration methods because it is convenient to use, does not increase the time beside the dental chair and has no discomfort.

Previous studies on the acceleration of OTM by SMF have used whole-body magnetic field exposure to establish animal models^34,35^. Since the magnetic field strength generated by magnets decreases exponentially with distance, the actual magnetic field strength delivered to the oral cavity is very limited, and the adverse effects of whole-body magnetic field exposure are unclear. To better align the experimental magnetic field exposure mode, the magnetic field action site and the magnetic field strength with those of clinical magnetic appliances, we used a small custom-made magnet to generate an SMF strength of 200 ± 20 mT, which is close to the clinical magnet strength of 200 ± 50 mT, and achieved localized magnetic field exposure in the oral cavity. Thus, our local rat SMF exposure model has the advantage of being consistent with the clinical application environment. In addition, previous studies have shown that metallic elements produced by corroded magnets are cytotoxic and require encapsulation for biosafety^36^. To achieve isolation of the magnets from saliva, the NdFeB magnets used in our experiments were plated and encapsulated to increase their corrosion resistance and were encapsulated in a resin designed for oral use with good biosafety. Preliminary indications by body weight measurement showed no significant effect on the general health of the rats. Therefore, our model has the advantages of high biosafety, compactness, comfort, and sparing of the oral function and health of the animals.

Osteoclast resorption of bone is the initiation stage of OTM. Previous studies found that macrophages cultured in vitro in the presence of an SMF of 200 mT increased osteoclast differentiation^37^. It has also been found that SMF can reduce dental bone transparency and increase the thickness of the periodontal membrane^34^. In addition to finding an increase in osteoclasts on the pressure side and a greater distance of tooth movement under localized SMF exposure, our study also revealed a smaller unit of bone volume on the pressure side. This provides new evidence for promoting osteoclast function during SMF-enhanced tooth movement compared with previous studies. One SMF exposure of 0.2–0.4 T has been reported to reduce osteoclasts in mouse bone trabeculae^38^. The reason for the difference may be that the animal model is different, the former was the removal of mechanical stimulation, but our study was the under mechanical loading.

PDLSCs are important effector cells in the OTM process and are mechanosensitive cells that can convert mechanical, physical stimuli into chemical signals, causing changes in relevant signaling pathways and triggering various physiological processes^39^. PDLSCs respond to stress with altered biological properties and release several cytokines, including TNF-α, IL-1, and IL-6, through paracrine pathways involved in bone remodeling in OTM^40^. Our previous study showed that SMF stimulates PDLSC proliferation, migration, differentiation, and activation through the AKT pathway, indicating that PDLSCs can respond to SMF stimulation^21^. We performed the first transcriptomic analysis of PDLSCs under SMF and pressure stimulation, and the observation of many DEGs clarified the existence of a regulatory role of SMF in PDLSC physiology. In our experiments although a trend of elevated IL-6 expression was found on the Mech. loading + SMF group compared to the Mech. loading group, no statistically significant difference was observed. We speculate that cellular heterogeneity of primary cells from different individuals is an important reason for this phenomenon^41^. The insufficient number of samples may also be one of the reasons.

The OTM process is an aseptic inflammatory bone remodeling process, and inhibition of inflammation during the period of tooth movement reduces the rate of OTM^42^. According to our study, inflammatory factors like TNF-α and IL-1β had their expression levels reduced by SMF, but IL-6 had an increased outcome. This is consistent with the finding that SMF promotes the elevation of IL-6 in microglia but inconsistent with the finding that SMF inhibits the reduction of IL-6 levels in LPS-stimulated microglia^11^. We believe that this difference is related to the fact that animal models and models of inflammation are different, our study was on sterile inflammation induced by mechanical stimulation, unlike LPS-induced inflammation.

Our study found significantly elevated IL-6 expression and increased osteoclasts in SMF-exposed, stress-loaded cell and animal models, suggesting that IL-6 plays an essential role in OTM. One study elevated blood levels of IL-6 after orthodontic surgery. IL-6 regulates osteoclast formation by regulating the secretion of cytokines such as RANKL. Moreover, *IL-6* knockout mice exhibited reduced osteoclasts^43^, and IL-6 binds to IL-6 receptor induced significant osteoclast formation, suggesting a close relationship between this cytokine and osteoclast formation^44^. In an *in-vitro* model, we found that SMF potentiated IL-6 secretion by PDLSCs under mechanical stress, and we speculate that SMF promotes OTM by promoting a high expression of IL-6 on the stress side of the tooth and by maintaining the inflammatory microenvironment required for OTM on the stress side to stimulate osteoclast formation.

Tocilizumab is a commercially available IL-6 inhibitor, and previous studies have shown that inhibition can be achieved by local injection^45^. We used tocilizumab for local injection in a pressure-loaded experimental model of SMF exposure and observed that it significantly inhibited the effect of SMF in promoting OTM. However, the distance of OTM remained greater than that of the OTM + NaCl group, suggesting that SMF promotes OTM through additional signaling and molecular effects that will require in-depth investigation. Orthodontic bone remodelling is a complex aseptic inflammatory response process involving numerous inflammatory factors and immune cells, etc. IL-6 is one of the participants in mechanical force-induced tooth movement, and there are other factors that synergise in a complex environment in vivo. In addition, IL-6 was not elevated proportionally in the Mech. loading group as in the Mech. loading + SMF group, and SMF promoted IL-6 expression. The effect of the inhibitor was more obvious in the OTM + SMF group with the largest difference in IL6 expression. Therefore, tooth movement in the OTM group was inhibited to some extent after inhibitor administration but not as significantly as in the Mech. loading + SMF group.

In summary, our study clarifies that SMF can promote OTM. SMF may promote IL-6 secretion from PDLSCs through paracrine effects while reducing the production of TNF-α and IL-1 and other inflammatory molecules. The aseptic inflammatory microenvironment required to maintain OTM on the stress side can stimulate osteoclast formation to accelerate OTM. The results of this study are important for elucidating the mechanism of SMF in promoting OTM and give a biological foundation and explanation for the clinical use of SMF for accelerating OTM. In addition, this study has important implications for the precise regulation of tooth mobility, suggesting the use of SMF to accelerate the movement of the target tooth while using IL-6 inhibitors to reduce the undesired movement of another tooth. We thus provide a new OTM control concept for orthodontists to control different tooth movements precisely.

## Conclusion

SMF can promote tooth movement in rats. The SMF promotes IL-6 secretion from PDLSCs in the presence of mechanical pressure, and inhibition of IL-6 signaling can significantly attenuate the accelerating effect of SMF on tooth movement.

## Materials and methods

### Animals

All the animal experiments were approved by the Ethics Committee of the Animal Laboratory of Kunming Medical University (no. Kmmu20220137). The Laboratory Animal Centre at Kunming Medical University provided specific-pathogen-free, 8-week-old, healthy male Sprague–Dawley rats. The rats were maintained in a barrier environment throughout the experiments and fed conventional food and water. Moreover, frequent body weight measurements were made regularly. The testing was conducted in accordance with the ARRIVE recommendations, the U.K. Animals (Scientific Procedures) Act, 1986 and related regulations, EU Directive 2010/63/EU for animal experiments, or the National Institutes of Health's manual for the use and care of laboratory animals (NIH Publications No. 8023, revised 1978).

### OTM model

Rats were anesthetized with 3% sodium pentobarbital (0.1 mL/100 g) by intraperitoneal injection. Retention grooves approximately 0.1-mm deep were ground into the mesial surface of the left maxillary first molar and into both sides of the maxillary incisors. A 4-mm-long NiTi constant-force tension spring (50 g, SuHang, Shenzhen, China) was fixed between the left upper first molar and the maxillary incisors with a 0.20-mm metal-wire ligature. The tension spring was adjusted using a spring dynamometer to produce a horizontal force of 50 g before fixation. Light-cured resin (3M, St. Paul, MN, USA) was used to reinforce the upper-incisor ligature. The model did not demold during the experiments.

### Localized magnetic-field exposure model

While the OTM model was being established, the OTM + SMF group received a 5-0 nylon suture (Lingqiao, Ningbo, China) to fix a NdFeB magnet (Yuexing Magnet Co., Shenzhen, China) to the left buccal mucosa of the rat opposite the first molar. The magnets were 4 mm in diameter and 1-mm high, with a 1.5-mm-diameter circular hole in the center and covered with a biosafe resin (3M, St. Paul, MN) to avoid possible cytotoxic damage. The OTM + sham group received 5-0 nylon sutures to fix a non-magnetic metal dummy with the same size and shape to the same position in the left cheek of the rats. The magnets and the non-magnetic metal-loaded dummy did not affect the normal functions of the rats, such as mastication. The magnetic field strength of the magnet was measured at 200 ± 20 mT using a Tesla Gaussmeter (Nohawk, Tianjin, China).

### IL-6 inhibitor injections

Tocilizumab (GlpBio, Montclair, CA) is an anti-IL-6R neutralizing antibody that competitively binds IL-6R to inhibit the function of IL-6. Tocilizumab was diluted to 10 mg/mL with saline (Kelun, Sichuan, China) using a previously reported concentration^45^. The inhibitor group was injected with 50 μL of tocilizumab locally in the vestibular sulcus of the upper-left first molar, and the control group was injected with 50 μL of saline (Kelun, Sichuan, China) at the corresponding site. The injections were performed on days 1 and 4 of the experiment.

### Micro-CT analyses

Seven days after the models were established, the maxillary jaws and teeth were picked up and fixed in a 4% formaldehyde solution for 24 h. The maxillary jaws and tooth were scanned with a micro-computed tomography (micro-CT) system (PINGSENG Healthcare Inc., Kunshan, China). Images were reconstructed and analyzed using Mimics software 21.0 (Materialise, Leuven, Belgium). In the micro-CT image, the distance between the crowns of the first left two molars in the maxillary jaws was measured in a direction parallel to the direction of OTM as the distance of tooth movement. The OTM distance was determined by investigators blinded to the group.

### Histological analysis

After the CT scan, the rat maxillae were immersed in a 10% solution of EDTA (MVS-0098, MXB, FuZhou, China) for decalcification, and the solution was altered every 3 days. After 21–28 days, the tissue could be punctured without resistance with a sterile needle to establish the completion of decalcification. Afterward, the tissues were dehydrated in graded ethanols and embedded in paraffin. Four μm thick paraffin slices were cut and stained with hematoxylin and eosin (HE) and tartrate-resistant acid phosphatase (TRAP).

HE staining was used for histomorphological analysis. The sections were dewaxed and dehydrated, rinsed with distilled water, stained with hematoxylin for 15 min, rinsed with tap water, fractionated with 1% hydrochloric acid in alcohol for 2 s, rinsed, washed in phosphate-buffered saline (PBS), stained in eosin for 30 s, dehydrated, air-dried, and sealed with gum.

TRAP staining was used to identify and analyze osteoclasts. Sections were dewaxed, dehydrated, and incubated in TRAP solution (Sigma-Aldrich, St. Louis, MO) in accordance with the manufacturer’s instructions at 37 °C for 60 min, after which the osteoclasts appeared burgundy under the microscope. Sections were then stained with hematoxylin for 10 s, rinsed, fractionated with 1% hydrochloric acid in alcohol for 1 s, rinsed, dehydrated with 75% ethanol for 10 s, air-dried, and sealed with gum. The osteoclast area was analyzed using Image J-Pro plus 6.0 (National Institutes of Health, Bethesda, MD).

### Immunohistochemistry

After dewaxing and dehydration, the antigens in the EDTA-treated samples were heat-repaired in an oven at 100 °C for 40 min. Three percent hydrogen peroxide (SP KIT-A2, MXB, Fuzhou, China) was used to block the sections for 10 min and in goat serum for 45 min (SP KIT-B1, MXB, Fuzhou, China). Sections were then incubated with IL-6 primary antibody (1:400; GXP263019, Genxspan, Alabama, USA) overnight at 4 °C for approximately 10 h. Slices were then washed with PBS (Hyclone, Cytiva, Marlborough, MA, USA) and incubated with a secondary antibody (KIT-5010, Maishin, Fuzhou, China) for 15 min at 37 °C. Nuclei were re-stained with hematoxylin (DAB0031, MXB, Fuzhou, China) for 30 s, dehydrated, and sealed in gum. For semi-quantitative analysis, the average optical density (AOD) of IL-6 in the stained tissues was calculated using Image J-Pro plus 6.0 (National Institutes of Health, Bethesda, MD). AOD = (integrated optical density)/area.

### Isolation and culture of periodontal stem cells

This part of the experiment was approved by the Ethics Committee of the Affiliated Stomatological Hospital of Kunming Medical University (No. KYKQ2022MEC030), and informed consent was obtained from the patients in accordance with the Declaration of Helsinki. PDLSCs were isolated and cultured as previously described^46^. Briefly, PDLSCs were taken from the premolars of young patients 12–20 years old. These premolars had been removed for orthodontic treatment and were healthy teeth without dental or periodontal disease. Primary PDLSCs were isolated from these premolars and cultured after enzymic digestion in α-MEM medium (Hyclone, Logan, Utah) with 15% fetal bovine serum (FBS; BI, Israel) and 100 U/mL penicillin/streptomycin (Hyclone) for 7 days at 37 °C in a humidified environment with 5% CO_2_. When cell density reached ≥ 70%, the cells were purified, expanded, and subjected to limiting dilution analysis.

### Cellular additive force model

P5-generation PDLSCs in good condition were inoculated into 6-well plates (Corning Incorporated, Corning, NY, USA) at 2 × 10^5^ cells per well and pressure-loaded when cell confluence reached 80%. A 33-mm-diameter, 17 g transparent glass plate (Qingxing Glass, Shenzhen, China) was placed flat on the cell layer, in uniform contact with the cell layer, exerting a pressure of 2 g/cm^2^ on the cells. The PDLSCs were subjected to stress continuously for 24 h, followed by collecting the conditioned medium.

### Cellular magnetic-field exposure model

Cells were stimulated by a customized NdFeB SMF device (Li Tian Magnetics Technology Co., Ltd., Sichuan, China), which could generate a magnetic field with a strength of 200 ± 50 mT. Cells were exposed to the magnetic field for 24 h, and then the conditioned medium was collected. All plates were incubated in the same incubator.

### Extraction and culture of bone marrow macrophages (BMMs)

BMMs were extracted from 6-week-old C57 mice tibias and femurs. Monocytes were raised in α-MEM medium with 10% FBS and 25 ng/mL of M-CSF (CB34, Novoprotein, Shanghai), and nonadherent cells were washed after 2 days to obtain macrophages. Macrophages were cultured in PDLSC-conditioned medium and a-MEM containing 10% FBS (BI, Israel), and thereafter with osteoclast induction solution containing 100 ng/mL of RANKL (CR06, Novoprotein, Shanghai) and 25 ng/mL of M-SCF. TRAP staining was used to detect osteoclasts after 5–7 days.

### Western blot

Before immunoblotting, protein content was determined using the assay kit (BL521A, White Shark, Hefei, China), and all samples were adjusted to the same protein concentration. To separate equal quantities of protein, 12.5% polyacrylamide-gel electrophoresis was used. After electrophoresis, polyvinylidene fluoride membranes (Millipore, Billerica, MA, USA) were used to transport proteins. Next, the membranes were incubated with various primary antibodies, including TRAP (1:1000; #13908, CST, Germany), and b-tubulin (1:20,000; Proteintech, Rosemont, IL, USA), following treatment with a secondary antibody labeled with horseradish peroxidase (goat anti-rabbit, 1:5000, CST, Germany). Protein bands were detected using an electrochemiluminescence detection kit (P0018, Biyuntian, Shanghai, China) and an electrochemical gel imaging system (ChemiDocTM XPS+; Bio-Rad, Hercules, CA, USA). b-Tubulin expression was used as an internal reference.

### siRNA transfection

GenePharma (Shanghai, China) supplied the small interfering RNAs (siRNAs). The RNA sequences are provided in Supplementary Table 1. IL6-siRNA or siNC was transfected into hPDLSCs (70% confluent) in accordance with the manufacturer’s instructions (Lipofectamine™ 2000 Transfection Reagent, Invitrogen, USA). The transfection efficiency was calculated by observing the number of positively expressed cells under a confocal scanning microscope after 24 h. Subsequently, a pressure of 2 g/cm^2^ was applied to the cells for 24 h and the conditioned medium was collected. The effect of interference was determined using RT-PCR.

### Real-time PCR

Cellular RNA was isolated using the Total RNA Extraction Kit (Tangan, Branford, CT, USA). We converted 1000 ng of RNA to cDNA using the PrimeScript™ RT reagent Kit with the gDNA Eraser (RR047, Takara, Shiga, Japan). Real-time quantitative PCR was performed with TB Green^®^ Premix Ex Taq™ II (Tli RNaseH Plus) (RR820, Takara). Amplification conditions were as follows: 95 °C for 30 s, 40 cycles at 95 °C for 5 s, and 60 °C for 34 s. Relative gene expression levels were quantified using the 2^−ΔΔCt^ method. The mRNA expressions of the target genes were normalized to that of b-actin. The primer sequences are provided in Supplementary Table 2.

### RNA-seq analysis

The PDLSCs were exposed to an SMF and mechanical loading for 24 h. RNA was then obtained using the above technique. The quality of the RNA was evaluated on a 2100 Expert Bioanalyzer (Agilent, Santa Clara, CA, USA). Shanghai Majorbio Bio-Pharm Technology selected qualified RNA samples for library creation and sequencing on the NovaSeq 6000 platform (Illumina, San Diego, CA, USA). The Majorbio Cloud Platform (http://www.Majorbio.com), a free online platform, was used to analyze and visualize the data. Differential expression analysis was performed using the RSEM, DEGs with |log2FC| ≥ 1 and FDR < 0.05 (DESeq2) were considered to be significantly different expressed gene. We have uploaded the raw RNA-seq dataset to the NCBI, the accession number is PRJNA1099930.

### Statistical analysis

SPSS 25.0 was used for the statistical analysis. The raw data were confirmed to have a normal distribution using a one-sample Kolmogorov–Smirnov test. The independent *t*-test was used to compare the two groups. To compare three or more groups, a one-way variance analysis was employed, followed by Dunnett’s post-hoc multiple comparisons. Each datum represents at least three independent experiments. The results are presented as means ± standard deviation (SD). Differences with P < 0.05 were considered statistically significant.

## Supplementary Information

Supplementary Information.

## Data Availability

All other data generated and/or analysed during the current study are included in this article.

## Abbreviations

PDLSCs: Human periodontal ligament stem cells
BMMs: Bone marrow macrophages
SMF: Static magnetic field
OTM: Orthodontic tooth movement
IL: Interleukin
DEGs: Differential expressed genes
Mech. loading: Mechanical loading
TRAP: Tartrate-resistant acid phosphatase
BV: Bone volume
TV: Total volume
Oc.S: Osteoclast surface
B.S: Bone surface

## References

[1] National Health Institute of Hospital Administration, The Experts, Group of the Project of Standard Diagnose and Treatment Protocols for Early Orthodontic Interventions of Malocclusions of Children *et al.* China experts’ consensus on preventive and interceptive orthodontic treatments of malocclusions of children. *Hua Xi Kou Qiang Yi Xue Za Zhi* **39**(4), 369–376. 10.7518/hxkq.2021.04.001 (2021). 34409791 10.7518/hxkq.2021.04.001 PMC8381117

[2] Krishnan, V. & Davidovitch, Z. Cellular, molecular, and tissue-level reactions to orthodontic force. *Am. J. Orthod. Dentofacial Orthop.* **129**(4), 469 e461–432. 10.1016/j.ajodo.2005.10.007 (2006). 10.1016/j.ajodo.2005.10.007 16627171

[3] Ren, Y., Jongsma, M. A., Mei, L., van der Mei, H. C. & Busscher, H. J. Orthodontic treatment with fixed appliances and biofilm formation—A potential public health threat?. *Clin. Oral Investig.* **18**(7), 1711–1718. 10.1007/s00784-014-1240-3 (2014). 24728529 10.1007/s00784-014-1240-3

[4] Segal, N. A. *et al.* Two configurations of static magnetic fields for treating rheumatoid arthritis of the knee: A double-blind clinical trial. *Arch. Phys. Med. Rehabil.* **82**(10), 1453–1460. 10.1053/apmr.2001.24309 (2001). 11588753 10.1053/apmr.2001.24309

[5] Taniguchi, N., Kanai, S., Kawamoto, M., Endo, H. & Higashino, H. Study on application of static magnetic field for adjuvant arthritis rats. *Evid. Based Complement. Alternat. Med.* **1**(2), 187–191. 10.1093/ecam/neh024 (2004). 15480444 10.1093/ecam/neh024 PMC516457

[6] Dellinger, E. L. A clinical assessment of the Active Vertical Corrector—A nonsurgical alternative for skeletal open bite treatment. *Am. J. Orthod.* **89**(5), 428–436. 10.1016/0002-9416(86)90075-8 (1986). 3458375 10.1016/0002-9416(86)90075-8

[7] Gianelly, A. A., Vaitas, A. S. & Thomas, W. M. The use of magnets to move molars distally. *Am. J. Orthod. Dentofacial Orthop.* **96**(2), 161–167. 10.1016/0889-5406(89)90257-6 (1989). 2756952 10.1016/0889-5406(89)90257-6

[8] Steger, E. R. & Blechman, A. M. Case reports: Molar distalization with static repelling magnets. Part II. *Am. J. Orthod. Dentofacial Orthop.* **108**(5), 547–555. 10.1016/s0889-5406(95)70056-0 (1995). 7484975 10.1016/s0889-5406(95)70056-0

[9] Shang, W. *et al.* Static magnetic field accelerates diabetic wound healing by facilitating resolution of inflammation. *J. Diabetes Res.* **2019**, 5641271. 10.1155/2019/5641271 (2019). 31886281 10.1155/2019/5641271 PMC6915019

[10] Hsieh, S. C. *et al.* Static magnetic field attenuates lipopolysaccharide-induced inflammation in pulp cells by affecting cell membrane stability. *ScientificWorldJournal* **2015**, 492683. 10.1155/2015/492683 (2015). 25884030 10.1155/2015/492683 PMC4391652

[11] Shen, L. K. *et al.* A static magnetic field attenuates lipopolysaccharide-induced neuro-inflammatory response via IL-6-mediated pathway. *Electromagn. Biol. Med.* **33**(2), 132–138. 10.3109/15368378.2013.794734 (2014). 23781996 10.3109/15368378.2013.794734

[12] Huang, H., Yang, R. & Zhou, Y. H. Mechanobiology of periodontal ligament stem cells in orthodontic tooth movement. *Stem Cells Int.* **2018**, 6531216. 10.1155/2018/6531216 (2018). 30305820 10.1155/2018/6531216 PMC6166363

[13] Zainal Ariffin, S. H., Yamamoto, Z., Zainol Abidin, I. Z., Megat Abdul Wahab, R. & Zainal Ariffin, Z. Cellular and molecular changes in orthodontic tooth movement. *ScientificWorldJournal* **11**, 1788–1803. 10.1100/2011/761768 (2011). 22125437 10.1100/2011/761768 PMC3201678

[14] He, D. *et al.* Mechanical load-induced H(2)S production by periodontal ligament stem cells activates M1 macrophages to promote bone remodeling and tooth movement via STAT1. *Stem Cell Res. Ther.* **11**(1), 112. 10.1186/s13287-020-01607-9 (2020). 32169104 10.1186/s13287-020-01607-9 PMC7071778

[15] Lee, S. I. *et al.* Mechanical stress-activated immune response genes via Sirtuin 1 expression in human periodontal ligament cells. *Clin. Exp. Immunol.* **168**(1), 113–124. 10.1111/j.1365-2249.2011.04549.x (2012). 22385246 10.1111/j.1365-2249.2011.04549.x PMC3390502

[16] Manolagas, S. C. Role of cytokines in bone resorption. *Bone* **17**, S63–S67 (1995). 10.1016/8756-3282(95)00180-l 8579900

[17] Moon, S. J. *et al.* Temporal differential effects of proinflammatory cytokines on osteoclastogenesis. *Int. J. Mol. Med.* **31**(4), 769–777. 10.3892/ijmm.2013.1269 (2013). 23403591 10.3892/ijmm.2013.1269 PMC3621814

[18] Mootoo, A., Stylianou, E., Arias, M. A. & Reljic, R. TNF-alpha in tuberculosis: A cytokine with a split personality. *Inflamm. Allergy Drug Targets* **8**(1), 53–62. 10.2174/187152809787582543 (2009). 19275693 10.2174/187152809787582543

[19] O’Brien, C. A. STAT3 activation in stromal/osteoblastic cells is required for induction of the receptor activator of NF-kappaB ligand and stimulation of osteoclastogenesis by gp130-utilizing cytokines or interleukin-1 but not 1,25-dihydroxyvitamin D3 or parathyroid hormone. *J. Biol. Chem.* **274**, 19301–19308 (1999). 10383440 10.1074/jbc.274.27.19301

[20] Tamura, T. *et al.* Soluble interleukin-6 receptor triggers osteoclast formation by interleukin 6. *Proc. Natl. Acad. Sci. USA* **90**, 11924–11928 (1993). 8265649 10.1073/pnas.90.24.11924 PMC48097

[21] Zhang, K., Ge, W., Luo, S., Zhou, Z. & Liu, Y. Static magnetic field promotes proliferation, migration, differentiation, and AKT activation of periodontal ligament stem cells. *Cells Tissues Organs*10.1159/000524291 (2022). 35344952 10.1159/000524291 PMC10534995

[22] Mayr, A. *et al.* Autophagy induces expression of IL-6 in human periodontal ligament fibroblasts under mechanical load and overload and effects osteoclastogenesis in vitro. *Front. Physiol.* **12**, 716441. 10.3389/fphys.2021.716441 (2021). 34512388 10.3389/fphys.2021.716441 PMC8430222

[23] Padisar, P., Hashemi, R., Naseh, M., Nikfarjam, B. A. & Mohammadi, M. Assessment of tumor necrosis factor alpha (TNFα) and interleukin 6 level in gingival crevicular fluid during orthodontic tooth movement: A randomized split-mouth clinical trial. *Electron. Physician* **10**(8), 7146–7154. 10.19082/7146 (2018). 30214696 10.19082/7146 PMC6122871

[24] Almpani, K. & Kantarci, A. Surgical methods for the acceleration of the orthodontic tooth movement. *Front. Oral Biol.* **18**, 92–101. 10.1159/000382051 (2016). 26599122 10.1159/000382051

[25] Benjakul, S., Jitpukdeebodintra, S. & Leethanakul, C. Effects of low magnitude high frequency mechanical vibration combined with compressive force on human periodontal ligament cells in vitro. *Eur. J. Orthod.* **40**(4), 356–363. 10.1093/ejo/cjx062 (2018). 29016746 10.1093/ejo/cjx062

[26] Benjakul, S., Unat, B., Thammanichanon, P. & Leethanakul, C. Vibration synergistically enhances IL-1beta and TNF-alpha in compressed human periodontal ligament cells in the frequency-dependent manner. *J. Oral Biol. Craniofac. Res.* **10**(4), 412–416. 10.1016/j.jobcr.2020.06.005 (2020). 32775184 10.1016/j.jobcr.2020.06.005 PMC7397387

[27] Phusuntornsakul, P., Jitpukdeebodintra, S., Pavasant, P. & Leethanakul, C. Vibration enhances PGE(2), IL-6, and IL-8 expression in compressed hPDL cells via cyclooxygenase pathway. *J. Periodontol.* **89**(9), 1131–1141. 10.1002/jper.17-0653 (2018). 29761497 10.1002/JPER.17-0653

[28] Fernandes, M. R. U., Suzuki, S. S., Suzuki, H., Martinez, E. F. & Garcez, A. S. Photobiomodulation increases intrusion tooth movement and modulates IL-6, IL-8 and IL-1beta expression during orthodontically bone remodeling. *J. Biophotonics* **12**(10), e201800311. 10.1002/jbio.201800311 (2019). 31001928 10.1002/jbio.201800311

[29] Isola, G., Ferlito, S. & Rapisarda, E. Low-level laser therapy increases interleukin-1beta in gingival crevicular fluid and enhances the rate of orthodontic tooth movement. *Am. J. Orthod. Dentofacial Orthop.* **155**(4), 456–457. 10.1016/j.ajodo.2019.01.004 (2019). 30935594 10.1016/j.ajodo.2019.01.004

[30] Karabel, M. A., Dogru, M., Dogru, A., Karadede, M. I. & Tuncer, M. C. Evaluation of the effects of diode laser application on experimental orthodontic tooth movements in rats. Histopathological analysis. *Acta Cir. Bras.* **35**(12), e351204. 10.1590/ACB351204 (2021). 33503217 10.1590/ACB351204 PMC7819686

[31] Tsuka, Y. *et al.* Examination of the effect of combined use of Er:YAG laser irradiation and mechanical force loading on bone metabolism using primary human gingival fibroblasts. *Lasers Med. Sci.* **35**(9), 2059–2064. 10.1007/s10103-020-03079-y (2020). 32577932 10.1007/s10103-020-03079-y

[32] Zheng, J. & Yang, K. Clinical research: Low-level laser therapy in accelerating orthodontic tooth movement. *BMC Oral Health* **21**(1), 324. 10.1186/s12903-021-01684-z (2021). 34182967 10.1186/s12903-021-01684-z PMC8237464

[33] Lew, W. Z., Feng, S. W., Lee, S. Y. & Huang, H. M. The review of bioeffects of static magnetic fields on the oral tissue-derived cells and its application in regenerative medicine. *Cells*10.3390/cells10102662 (2021). 34685642 10.3390/cells10102662 PMC8534790

[34] Shan, Y. *et al.* The effects of static magnetic field on orthodontic tooth movement in mice. *Bioelectromagnetics* **42**(5), 398–406. 10.1002/bem.22346 (2021). 34033679 10.1002/bem.22346

[35] Chen, G. *et al.* Moderate SMFs attenuate bone loss in mice by promoting directional osteogenic differentiation of BMSCs. *Stem Cell Res. Ther.* **11**(1), 487. 10.1186/s13287-020-02004-y (2020). 33198804 10.1186/s13287-020-02004-y PMC7667787

[36] Guttal, S. S., Nadiger, R. K. & Shetty, P. Cytotoxic effect of indigenously fabricated dental magnets for application in prosthodontics. *J. Indian Prosthodont. Soc.* **18**(1), 29–34. 10.4103/jips.jips_114_17 (2018). 29430139 10.4103/jips.jips_114_17 PMC5799965

[37] Zhang, J. *et al.* Regulation of osteoclast differentiation by static magnetic fields. *Electromagn. Biol. Med.* **36**(1), 8–19. 10.3109/15368378.2016.1141362 (2017). 27355421 10.3109/15368378.2016.1141362

[38] Yang, J. *et al.* Static magnetic field of 0.2–0.4 T promotes the recovery of hindlimb unloading-induced bone loss in mice. *Int. J. Radiat. Biol.* **97**(5), 746–754. 10.1080/09553002.2021.1900944 (2021). 33720796 10.1080/09553002.2021.1900944

[39] Jin, S. S. *et al.* Mechanical force modulates periodontal ligament stem cell characteristics during bone remodelling via TRPV4. *Cell Prolif.* **53**(10), e12912. 10.1111/cpr.12912 (2020). 32964544 10.1111/cpr.12912 PMC7574874

[40] Li, Y., Zhan, Q., Bao, M., Yi, J. & Li, Y. Biomechanical and biological responses of periodontium in orthodontic tooth movement: Up-date in a new decade. *Int. J. Oral Sci.* **13**(1), 20. 10.1038/s41368-021-00125-5 (2021). 34183652 10.1038/s41368-021-00125-5 PMC8239047

[41] Cui, W. *et al.* High heterogeneity undermines generalization of differential expression results in RNA-Seq analysis. *Hum. Genom.* **15**(1), 7. 10.1186/s40246-021-00308-5 (2021). 10.1186/s40246-021-00308-5 PMC7845028 33509298

[42] Bartzela, T., Turp, J. C., Motschall, E. & Maltha, J. C. Medication effects on the rate of orthodontic tooth movement: A systematic literature review. *Am. J. Orthod. Dentofacial Orthop.* **135**(1), 16–26. 10.1016/j.ajodo.2008.08.016 (2009). 19121496 10.1016/j.ajodo.2008.08.016

[43] Wu, Q., Zhou, X., Huang, D., Ji, Y. & Kang, F. IL-6 enhances osteocyte-mediated osteoclastogenesis by promoting JAK2 and RANKL activity in vitro. *Cell Physiol. Biochem.* **41**(4), 1360–1369. 10.1159/000465455 (2017). 28278513 10.1159/000465455

[44] Feng, W., Yang, P., Liu, H., Zhang, F. & Li, M. IL-6 promotes low concentration of RANKL-induced osteoclastic differentiation by mouse BMMs through trans-signaling pathway. *J. Mol. Histol.* **53**(3), 599–610. 10.1007/s10735-022-10077-7 (2022). 35661290 10.1007/s10735-022-10077-7

[45] Zhou, R., Wu, X., Wang, Z., Ge, J. & Chen, F. Interleukin-6 enhances acid-induced apoptosis via upregulating acid-sensing ion channel 1a expression and function in rat articular chondrocytes. *Int. Immunopharmacol.* **29**(2), 748–760. 10.1016/j.intimp.2015.08.044 (2015). 26359543 10.1016/j.intimp.2015.08.044

[46] Liu, Y. *et al*. MiR-17 modulates osteogenic differentiation through a coherent feed-forward loop in mesenchymal stem cells isolated from periodontal ligaments of patients with periodontitis. *Stem Cells* **29**(11), 1804–1816. 10.1002/stem.728 (2011). 21898695 10.1002/stem.728

