# Correction: Static magnetic field-induced IL-6 secretion in periodontal ligament stem cells accelerates orthodontic tooth movement

**DOI:** 10.1038/s41598-025-22263-0

**Published:** 2025-10-13

**Authors:** Shitong Luo, Zhilian Li, Lizhiyi Liu, Juan Zhao, Wenbin Ge, Kun Zhang, Zhi Zhou, Yali Liu

**Affiliations:** 1 Department of Orthodontics, School and Hospital of Stomatology, Kunming Medical University, 1088 Middle Haiyuan Road, High-Tech Zone, Kunming, 650106 Yunnan China; 2 Yunnan Key Laboratory of Stomatology, Kunming, 650106 China; 3 Department of Orthodontics, Suining Central Hospital, Suining, 629000 China; 4 Department of Pathology, Suining Central Hospital, Suining, 629000 China; 5 Department of Orthodontics, Affiliated Hospital of Yunnan University, Yunnan University, 176 Qingnian Road, Wuhua District, Kunming, 650021 Yunnan China

Correction to: *Scientific Reports* 10.1038/s41598-024-60621-6, published online 29 April 2024

The original version of this Article contained errors.

As a result of an error during figure assembly, the Article contained an error in Fig. 3B, in which the images of Con group, Mech.loading group and Mech.loading+SMF group were duplicated. Consequently, the Figure 3 legend,

“SMF promoted osteoclast formation by PDLSCs under mechanical stress. (**A**) Schematic diagram of the in-vitro models. (**B**) Morphology of PDLSCs stimulated by mechanical loading and SMF after 24 h. Scale bar = 20 mm. (**C**) Schematic diagram of osteoclast formation induced by the PDLSCs supernatant. (**D**,**E**) Representative TRAP images showing osteoclast formation after 7 days of induction. (**F**,**G**) Western blot image and semi-quantitative measurement of TRAP expression in osteoclasts after 7 days of induction. The original images are shown in the Appendix. n = 3; ns, not significant; **P < 0.01 vs Con group; ^#^P < 0.05 vs Mech. loading group.”

now reads:

“SMF promoted osteoclast formation by PDLSCs under mechanical stress. (A) Schematic diagram of the in-vitro models. (B) Morphology of PDLSCs stimulated by mechanical loading and SMF after 24 h. (C) Schematic diagram of osteoclast formation induced by the PDLSCs supernatant. (D,E) Representative TRAP images showing osteoclast formation after 7 days of induction. (F,G) Western blot image and semi-quantitative measurement of TRAP expression in osteoclasts after 7 days of induction. n=3; ns, not significant; **P<0.01 vs Con group; #P<0.05 vs Mech. loading group.”

The original Figure 3 and accompanying legend appear below.

**Fig. 3:**
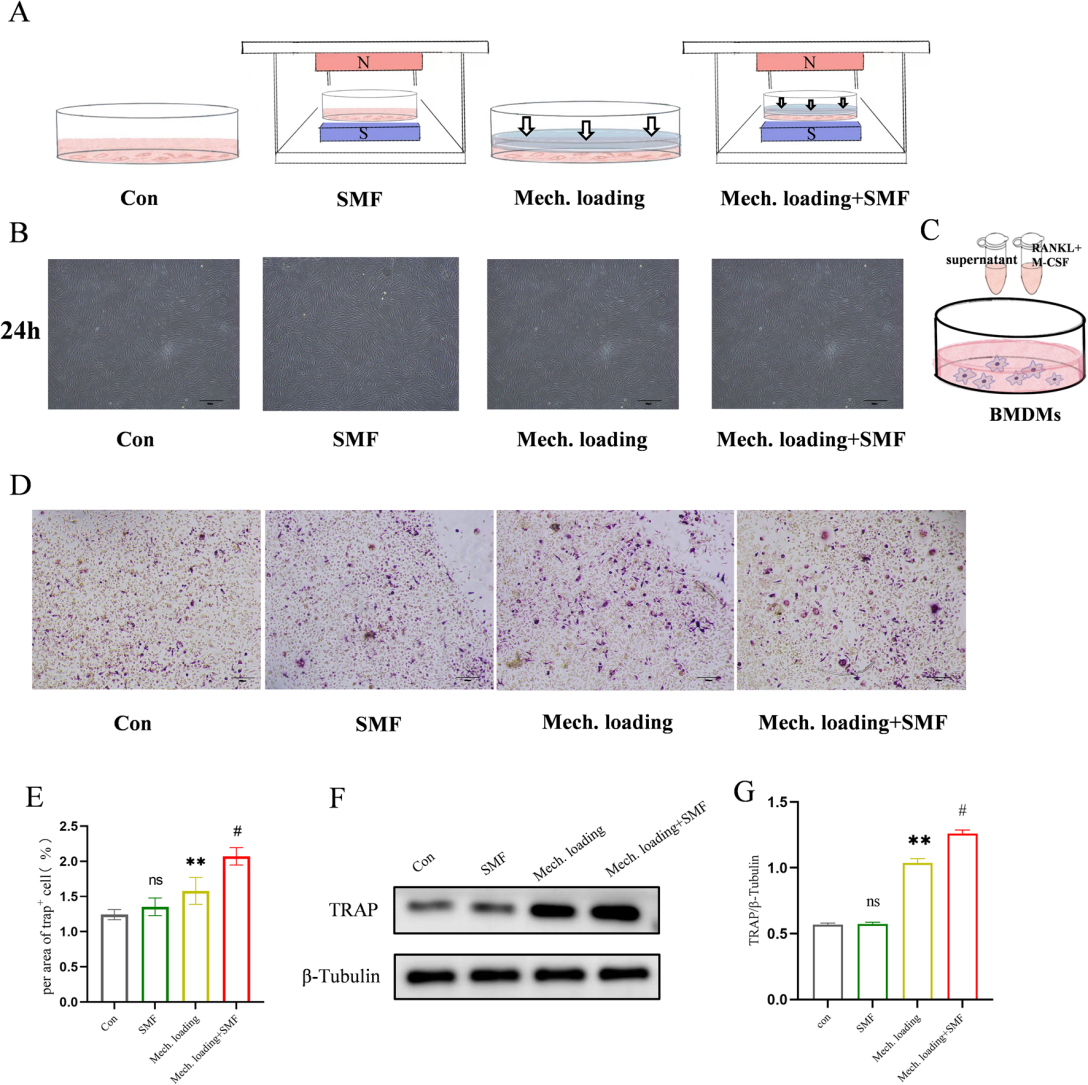
SMF promoted osteoclast formation by PDLSCs under mechanical stress. (**A**) Schematic diagram of the in-vitro models. (**B**) Morphology of PDLSCs stimulated by mechanical loading and SMF after 24 h. Scale bar = 20 mm. (**C**) Schematic diagram of osteoclast formation induced by the PDLSCs supernatant. (**D**,**E**) Representative TRAP images showing osteoclast formation after 7 days of induction. (**F**,**G**) Western blot image and semi-quantitative measurement of TRAP expression in osteoclasts after 7 days of induction. The original images are shown in the Appendix. n = 3; ns, not significant; **P < 0.01 vs Con group; ^#^P < 0.05 vs Mech. loading group.

Additionally, there was a duplication in Figure S4 from Figure 3D caused by improper data naming. Figure S4 was designed to verify the effect of each group of siRNA on IL-6 knockdown from the reagent company. All three groups of siRNA showed knockdown effect, and we selected the group with the most obvious knockdown effect for subsequent experiments.

The original Figure S4 and accompanying legend appear below.

**Fig. S4:**
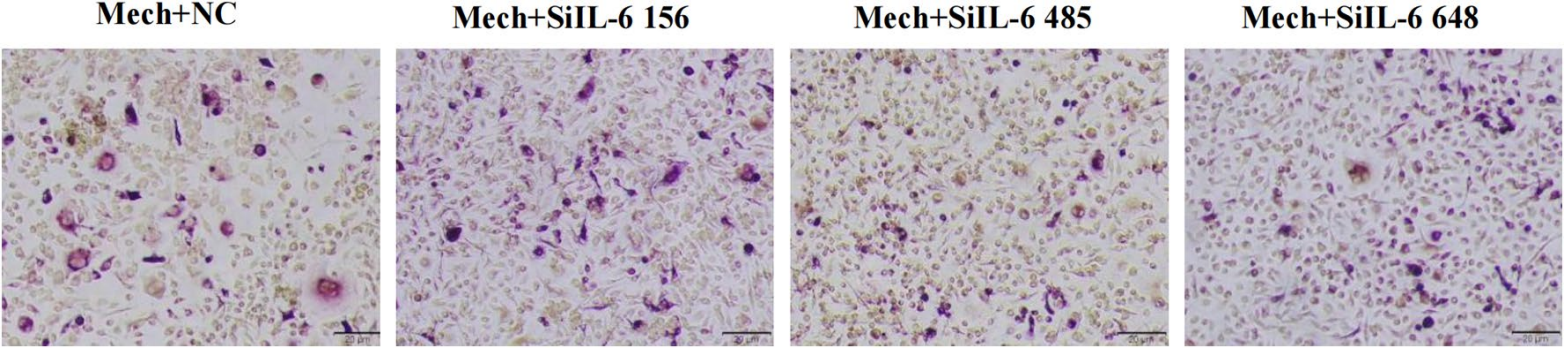
TRAP staining of osteoclast differentiation experiments using PDLSC supernatant after transfection knockdown of IL-6. The SiIL-6 485 group had the least number of TRAP positive cells.

The original Article and Supplementary Information file have been corrected.

