# Supplementary material for: Static magnetic field-induced IL-6 secretion in periodontal ligament stem cells accelerates orthodontic tooth movement
Source: Sci Rep. 2024 Apr 29;14:9851. doi: 10.1038/s41598-024-60621-6 (PMC11059396; doi:10.1038/s41598-024-60621-6)
Supplement: Supplementary file 1 — Supplementary Information. [file 41598_2024_60621_MOESM1_ESM.docx]

**Supplementary Materials**

**Static magnetic field-induced IL-6 secretion in periodontal ligament stem cells accelerates orthodontic tooth movement**

**Shitong Luo^1,2,3#^, Zhilian Li^1,2,#^, Lizhiyi Liu^1,2^,** **Juan Zhao^4^, Wenbin Ge^1,2^, Kun Zhang^1,2^, Zhi Zhou^5,^*, Yali Liu^1,2,^***

^1^ Department of Orthodontics, School and Hospital of Stomatology, Kunming Medical University, 1088 Middle Haiyuan Road, High-tech Zone, Kunming, Yunnan 650106, China.

^2^ Yunnan Key Laboratory of Stomatology, Kunming 650106, China.

^3^ Department of Orthodontics, Suining Central Hospital,Suining 629000, China.

^4^ Department of Pathology, Suining Central Hospital,Suining 629000, China.

^5^ Department of Orthodontics, Affiliated Hospital of Yunnan University, Yunnan University, 176 Qingnian Road, Wuhua District, Kunming, Yunnan 650021, China.

**Supplementary Table1**

| RNA | Sense 5′–3′ | Antisense (5′–3′) |
| --- | --- | --- |
| Si-NC | UUCUCCGAACGUGUCACGUTT | ACGUGACACGUUCGGAGAATT |
| SiIL6-156 | CAGGAGAAGAUUCCAAAGATT | UCUUUGGAAUCUUCUCCUGTT |
| SiIL6-485 | GCUGUGCAGAUGAGUACAATT | UUGUACUCAUCUGCACAGCTT |
| SiIL6-648 | GCAGCUUAAGGUUCCUTT | AGGAACUCCUUAAAGCUGCTT |

**Supplementary Table2**

Primer sequences for qRT-PCR.

| Gene (human) | Forward (5′–3′) | Reverse (5′–3′) |
| --- | --- | --- |
| GAPDH | CTTTGGTATCGTGGAAGGACTC | GTAGAGGCAGGGATGATGTTCT |
| IL-6 | CACTGGTCTTTTGGAGTTTGAG | GGACTTTTGTACTCATCTGCAC |
| IL-1β | GCCAGTGAAATGATGGCTTATT | AGGAGCACTTCATCTGTTTAGG |
| TNF-α | CCGCATCGCCGTCTCCTAC | CGCTGAGTCGGTCACCCTTC |
| OPG | GAAACGTTTCCTCCAAAGTACC | CTGTCTGTGTAGTAGTGGTCAG |
| RANKL | TTACCTGTATGCCAACATTTGC | TTTGATGCTGGTTTTAGTGACG |


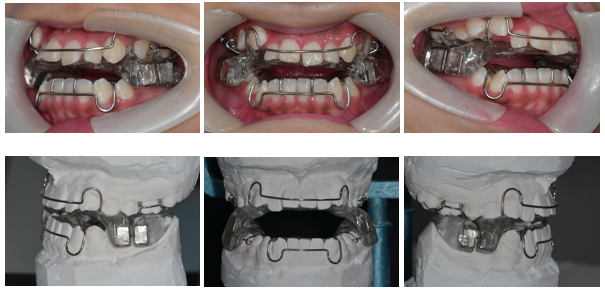
Figure S1. Clinical magnetic orthodontic appliances.


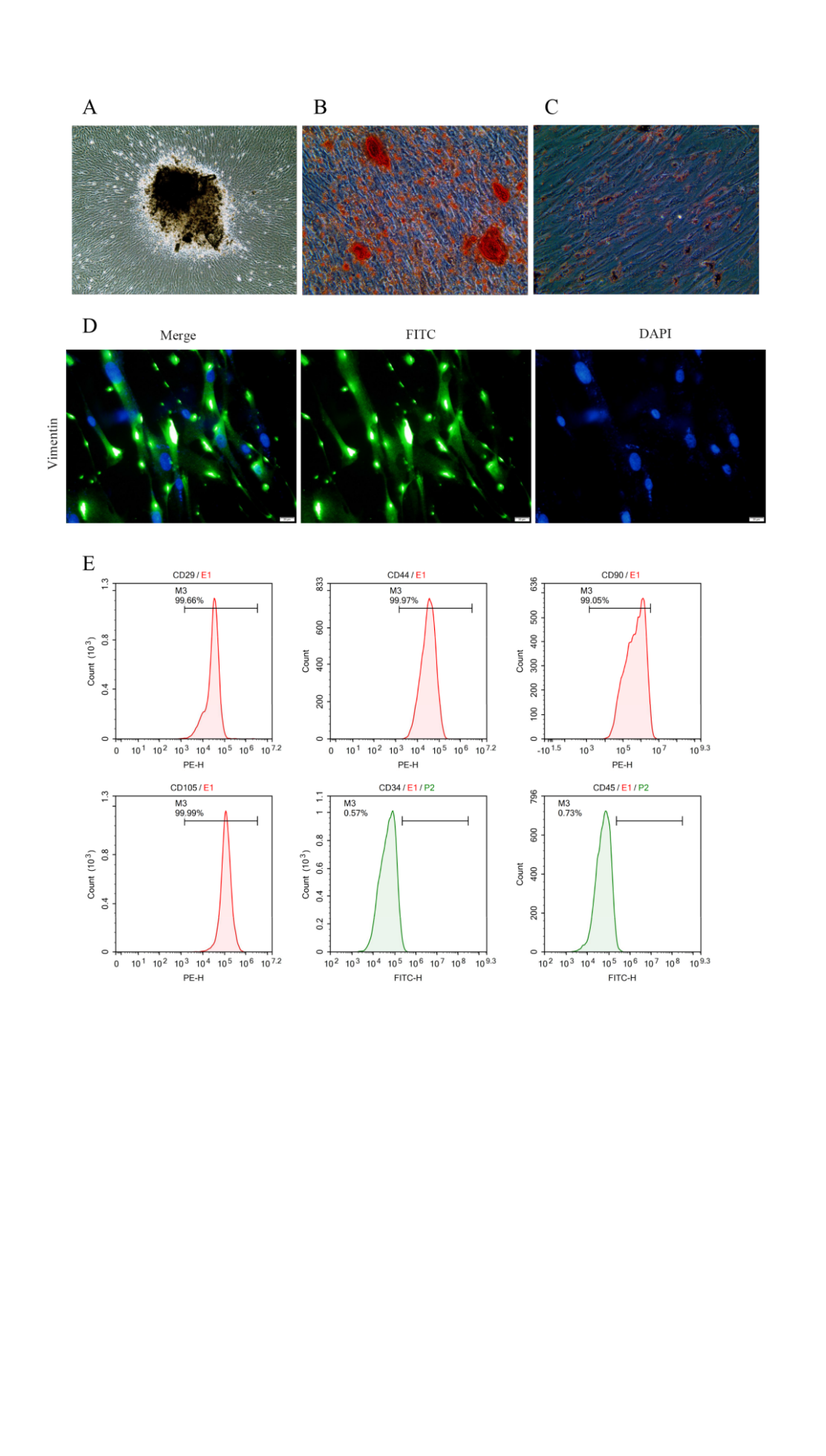


Figure S2. Culture and identification of PDLSCs. (A) Primary culture of PDLSCs was prepared by enzymatic tissue block method. Cells crawled out of the tissue block. (B) Alizarin red staining was used to detect the osteogenic differentiation of PDLSCs. (C) Oil red O staining was used to detect the adipogenic differentiation of PDLSCs. (D) Cellular immunofluorescence staining was used to detect markers of PDLSCs. (E) Flow cytometry was used to detect the markers of PDLSCs.


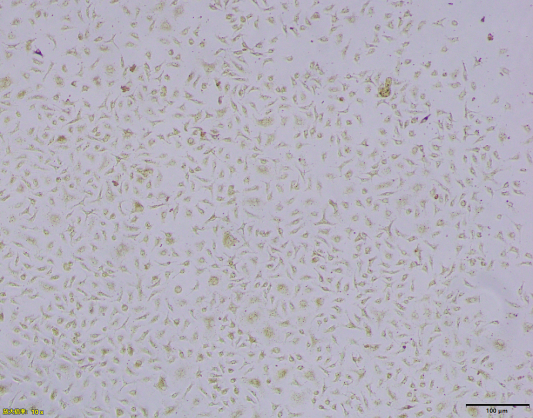


A

B


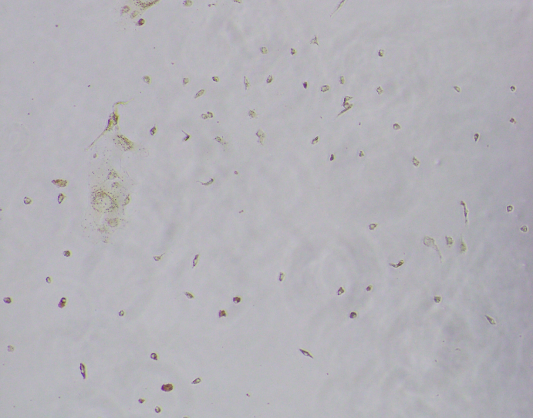

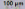


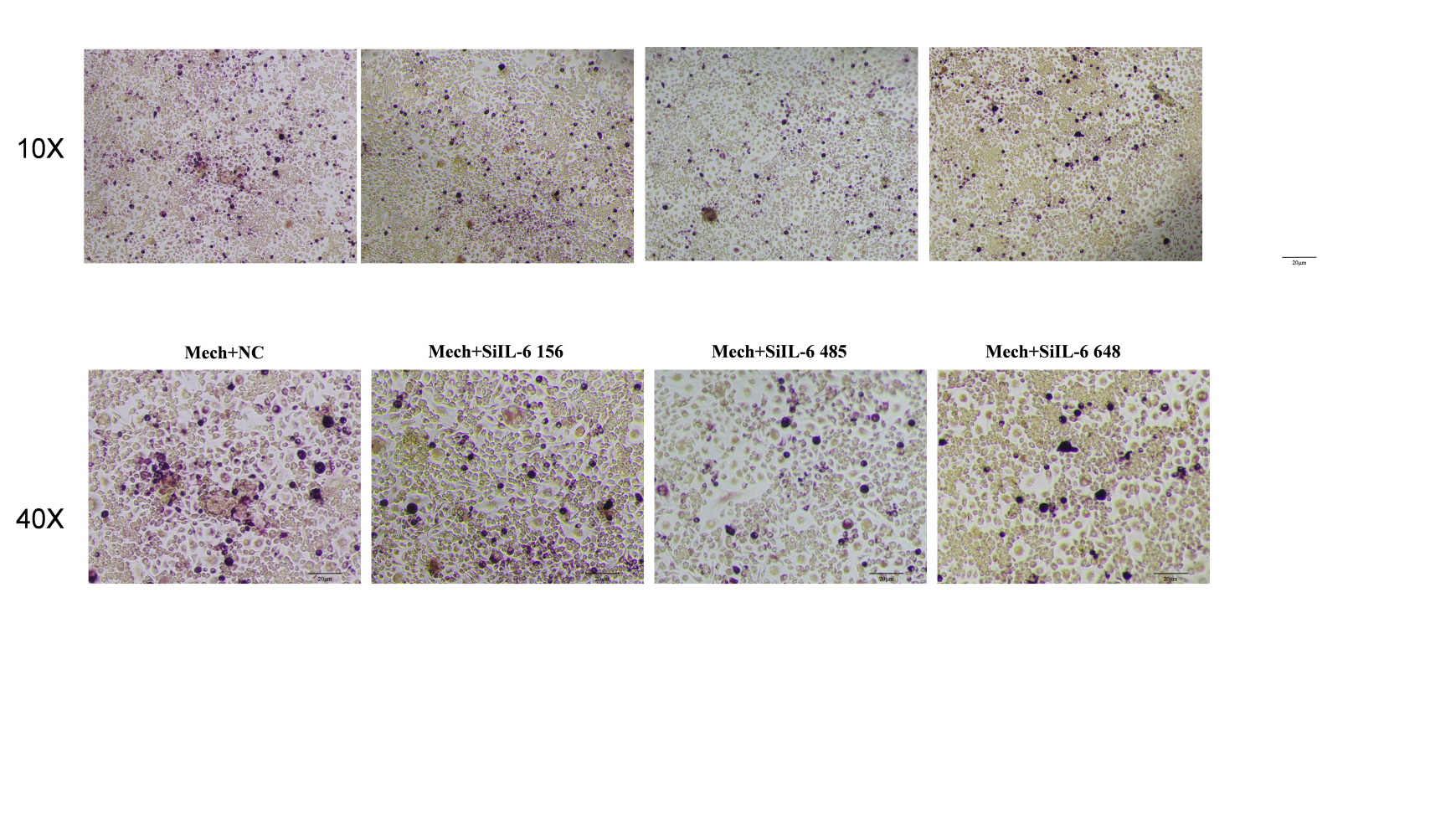
Figure S3. TRAP staining of osteoclast differentiation experiments using PDLSC supernatant. (A) RANKL, M-CSF (-) +Control group supernatant, almost all of the cells were not adherent. (B) RANKL, M-CSF (+) +Control group supernatant, There were almost no TRAP-positive osteoclasts.

Figure S4. TRAP staining of osteoclast differentiation experiments using PDLSC supernatant after transfection knockdown of IL-6. The SiIL-6 485 group had the least number of TRAP positive cells.


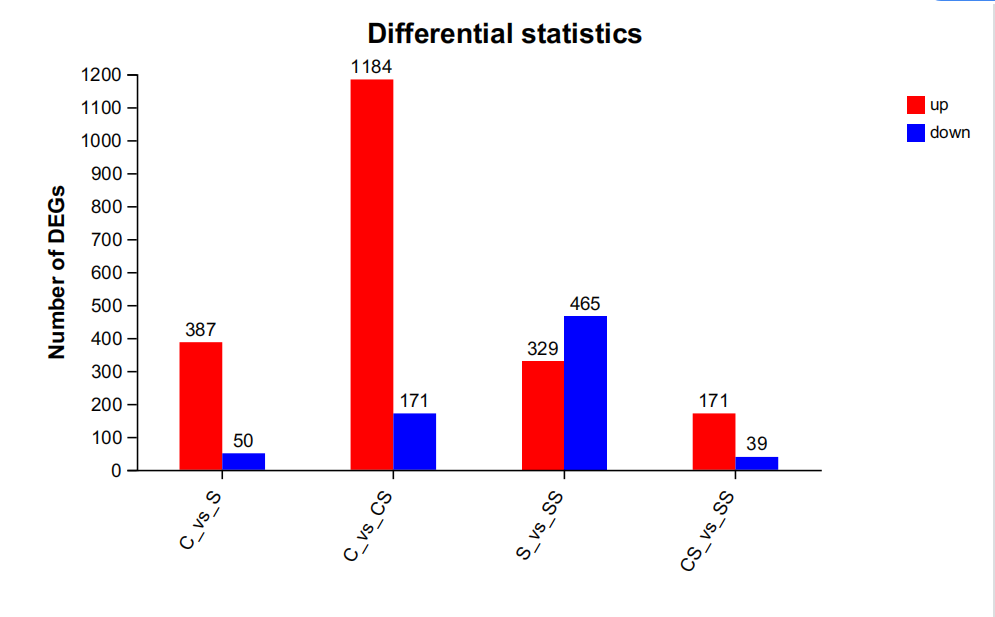


Figure S5. Statistical table of RNA-seq DEG. C=Con group, S=SMF group, CS=Mech. Loading group, SS=Mech. Loading+SMF group. Note: In C_-_V_-_S, up means the number of up-regulated genes in group S.


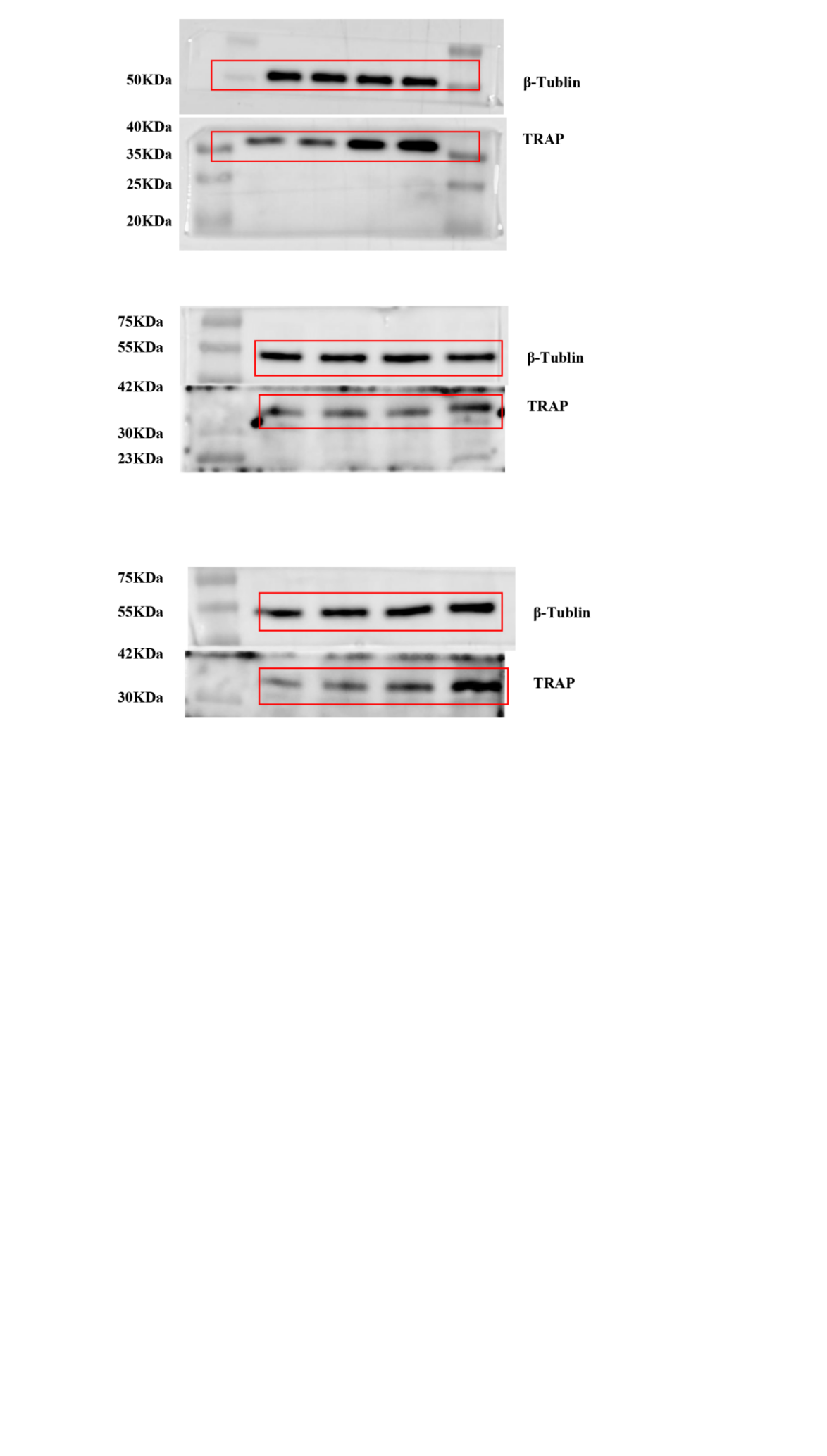

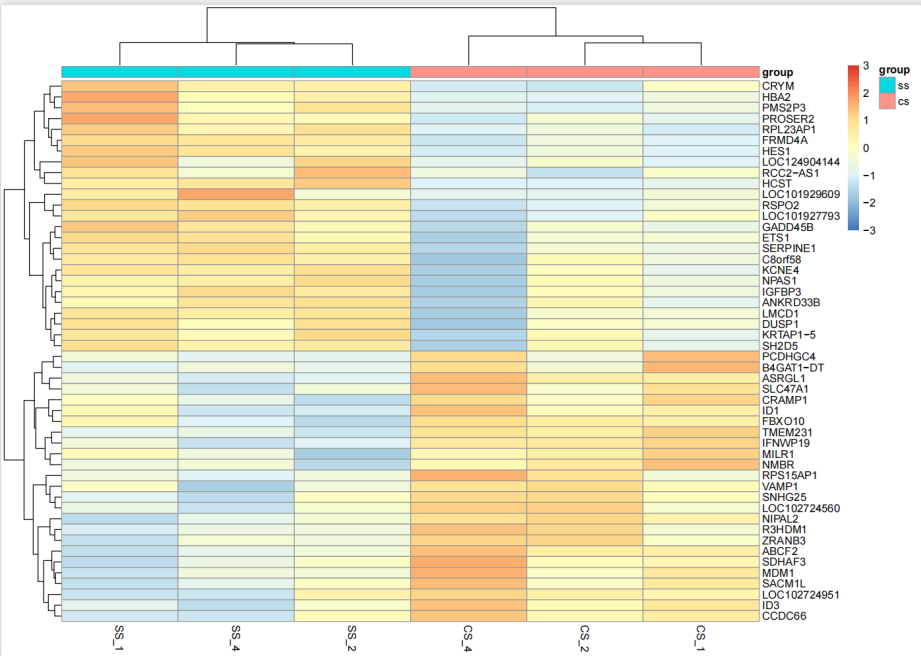
Figure S6: A:Heatmap of .Mech. Loading group and Mech. Loading+SMF group; B: Expression of IL-6 in the original data. N=3. CS=Mech. Loading group, SS=Mech. Loading+SMF group. 1, 2, 4 for patients 1, 2, 4.


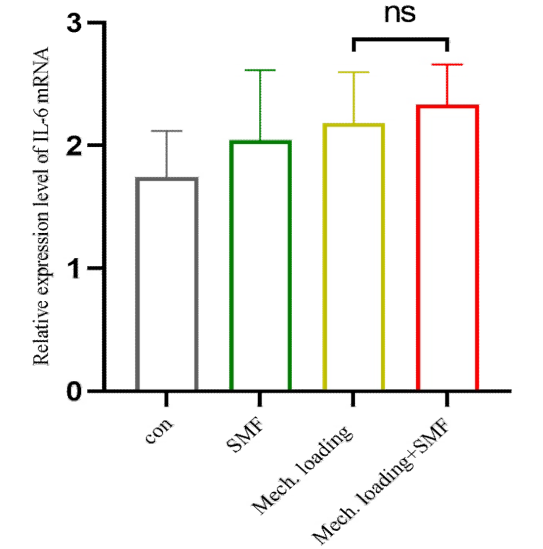


Figure S7. Full-size images of the Western blotting.
